# Migration towards Bangladesh coastlines projected to increase with sea-level rise through 2100

**DOI:** 10.1088/1748-9326/abdc5b

**Published:** 2021-02-10

**Authors:** A R Bell, D J Wrathall, V Mueller, J Chen, M Oppenheimer, M Hauer, H Adams, S Kulp, P U Clark, E Fussell, N Magliocca, T Xiao, E A Gilmore, K Abel, M Call, A B A Slangen

**Affiliations:** 1 Department of Earth and Environment, Boston University, Boston, MA 02215, United States of America; 2 Department of Environmental Studies, New York University, New York, NY 10012, United States of America; 3 College of Earth, Ocean and Atmospheric Sciences, Oregon State University, Corvallis, OR 97331-5503, United States of America; 4 School of Politics and Global Studies, Arizona State University, Tempe, AZ 85287-3902, United States of America; 5 International Food Policy Research Institute, Washington, DC 20005, United States of America; 6 Department of Agricultural, Environmental, and Development Economics, The Ohio State University, Columbus, OH 43210, United States of America; 7 School of Public and International Affairs and Department of Geosciences, Princeton University, Princeton, NJ 08544-1013, United States of America; 8 Department of Sociology, Florida State University, Tallahassee, FL 32306, United States of America; 9 Department of Geography, King's College London, London WC2R 2LS, United Kingdom; 10 Climate Central, Princeton, NJ 08542, United States of America; 11 School of Geography and Environmental Sciences, University of Ulster, Coleraine, Northern Ireland BT52 1SA, United Kingdom; 12 Population Studies and Training Center and the Institute at Brown on Environment and Society, Brown University, Providence, RI 02912, United States of America; 13 Department of Geography, University of Alabama, Tuscaloosa, AL 35401, United States of America; 14 Department of International Development, Community and Environment, Clark University, Worcester, MA 01610-1477, United States of America; 15 USAID, Washington, DC, United States of America; 16 Department of Estuarine and Delta Systems, NIOZ Royal Netherlands Institute for Sea Research, Yerseke 4401 NT, The Netherlands

**Keywords:** migration, sea-level rise, Bangladesh, agent-based model, trapped populations

## Abstract

To date, projections of human migration induced by sea-level change (SLC) largely suggest large-scale displacement away from vulnerable coastlines. However, results from our model of Bangladesh suggest counterintuitively that people will continue to migrate toward the vulnerable coastline irrespective of the flooding amplified by future SLC under all emissions scenarios until the end of this century. We developed an empirically calibrated agent-based model of household migration decision-making that captures the multi-faceted push, pull and mooring influences on migration at a household scale. We then exposed ~4800 000 simulated migrants to 871 scenarios of projected 21st-century coastal flooding under future emissions pathways. Our model does not predict flooding impacts great enough to drive populations away from coastlines in any of the scenarios. One reason is that while flooding does accelerate a transition from agricultural to non-agricultural income opportunities, livelihood alternatives are most abundant in coastal cities. At the same time, some coastal populations are unable to migrate, as flood losses accumulate and reduce the set of livelihood alternatives (so-called ‘trapped’ populations). However, even when we increased access to credit, a commonly-proposed policy lever for incentivizing migration in the face of climate risk, we found that the number of immobile agents actually rose. These findings imply that instead of a straightforward relationship between displacement and migration, projections need to consider the multiple constraints on, and preferences for, mobility. Our model demonstrates that decision-makers seeking to affect migration outcomes around SLC would do well to consider individual-level adaptive behaviors and motivations that evolve through time, as well as the potential for unintended behavioral responses.

Sea-level change (SLC)—including sea-level rise (SLR) and associated extreme sea levels—will drive human migration from vulnerable coastlines to the safer interior [1] by threatening the secure residence and livelihoods of coastal populations. The increased frequency of tidal extremes and flooding will cause permanent land loss due to submergence, episodes of temporary submergence due to flooding episodes, and rapid, but partially reversible, erosion. Additionally, rising salinity due to increased flooding and intrusion of seawater into groundwater will undermine agricultural productivity [2]. SLC is expected to amplify these coastal hazards and trigger involuntary migration, factor into individual and collective decisions about prospective, anticipatory migration, and in planned relocations away from the coast.

Current estimates of coastal migrants fail to capture the dynamic, complex relationships between migration and the gradual changes in the landscape caused by SLC [3, 4] creating gross oversimplifications of the migration process. Migration arises from complex processes involving the physical geography of coastlines and the social, economic, political, and demographic conditions in both coastal and inland areas to co-create the conditions of SLC migration. Destinations that first attracted migrants, for example, may repel them once labor and housing markets are saturated; and vulnerable communities may cease to send migrants after a critical mass has left, and is sending remittances to support income diversification [5]. In most areas of the world where SLC poses a threat to livelihoods, observational socioeconomic data are insufficient to observe these long-term behavioral dynamics. Even if data from historical analogues did exist, they may not be appropriate predictors of the future or serviceable in predictions across contexts, given the magnitude of projected SLC impacts.

Future projections of SLC driven migration must contend with a stalemate between a seemingly endless set of behavioral responses to SLC impacts and a lack of data on potential responses. Most estimates navigate this challenge by assuming a straightforward relationship between populations exposed to SLC and migration [6]. With few empirical studies quantifying migratory responses to SLC [2, 7, 8], numbers of potential coastal migrants are based on estimates of current populations residing in high-risk areas, such as low elevation coastal zones (LECZs) [6, 9] A more nuanced view acknowledging the heterogeneity of coastal populations assumes that people with social and economic assets to bear the costs of migration may be the first to migrate from high-risk areas, while those with fewer resources may endure SLC risk longer before migrating, if they do at all [10].

As a result, projections may underestimate the *immobility* of coastal populations. First of all, as SLC risk intensifies, a greater abundance of coastal livelihood opportunities in rural agricultural and urban labor markets may nevertheless attract migrants *toward* high-risk coastal areas, compared to safer, inland areas [11]. Some segments of society may be unable or unwilling to migrate as a form of adaptation [12, 13], becoming ‘trapped’ in place. This diverse group includes those who are wealth-constrained, lacking the financial capital required for migration investments or skills that are transferable at destinations, those with strong place attachment to their community, and those whose cultural rules underlie who can and cannot migrate. Currently, we also include in this group people who prefer to invest in coastal activities that reduce income risk [2]. For some, liquidity, i.e. access to credit, may be a major barrier to migration [14–16]. Yet it would be inappropriate to lump those that are wealth-constrained with those who have strong cultural attachments to place [13]. These aspects of immobility are rarely parsed and explored at scale due to the difficulty of capturing these social phenomena with existing data sources.

Together, the relative benefits of pro-coastal migration and the preference of coastal residents to adapt *in situ* support the notion that coastal populations may actually *grow* in vulnerable areas rather than dampen over time. To examine this possibility, we developed an agent-based model (ABM) that simulates dynamic, multifaceted migration decisions in Bangladesh and overcomes the methodological issues related to oversimplified SLC migration and erroneous assumptions surrounding ‘trapped’ populations. Our ABM allows us to integrate these competing migration pressures and evaluate the extent to which SLC should be assumed to redirect migration to inland areas. Further, our ABM distinguishes the contributions of economic constraints from social constraints on immobility by including a policy of credit provision to all agents during peak changes in exposure to SLC allowing us to evaluate some of the drivers of immobility. Using our ABM we test two key hypotheses. First, we hypothesize that SLC will produce net migration away from the coastlines over time [17]. Second, that a segment of the coastal population will also become less mobile over time due to compounding losses from SLC and constraints to credit.

## 1. Methods

To test whether flooding associated with future SLC will drive migration away from the coast (Hypothesis 1), we developed an application of the MIDAS (Migration, Intensification, and Diversification as Adaptive Strategies) ABM platform (supplementary information SI1–3 (available online at stacks.iop.org/ERL/16/024045/mmedia)) [18]. MIDAS simulates individual migration decisions, by calibrating their assessment of livelihood portfolios across space based on empirical observations, and modeling temporal outcomes over a range of SLC exposure scenarios.

### 1.1. MIDAS motivation

Earlier attempts to project SLC-induced migration have insufficiently linked SLC with the known underlying household mechanisms that influence migration, oversimplifying the complex human behaviors that result in compounding risk over time [19–21]. MIDAS is designed to simulate migration resulting from the multifaceted ways in which a uniform population responds to SLC risk over time, i.e. people’s individual disposition for migration, their access to resources and social networks, and their evolving perceptions of risk [6, 12, 22]. It is designed to represent migration as one among a full range of stationary livelihood activities available to households responding to climate impacts [2, 23, 24]. Among the small but growing number of ABMs applied to migration [e.g. 25–27], MIDAS is unique in allowing migration to emerge as a rare event from simultaneous consideration of livelihood opportunities and constraints at home and in alternative locations.

MIDAS builds on existing models implementing the push-pull-mooring theory of migration [28], in which income decisions are subject to push factors (e.g. lost income opportunities at home), pull factors (e.g. improved income opportunities and social network ties elsewhere), and moorings (e.g. credit constraints and land ownership in home locations). This approach complements current place-based modeling techniques such as gravity [29] or radiation [17] models, which are used to disentangle the relative importance of economies, labor markets and social networks. MIDAS takes an additional step by acknowledging the relative importance of individual place-utility effects on migration decisions, *viz.* an agent’s attachment to place [13], credit constraints [14, 15], and/or flood risk perception [30]. These effects are crucial for explaining why both mobility and immobility can simultaneously result under SLC exposure scenarios.

We summarize below the basic mechanisms of MIDAS and our experimental approach; complete descriptions, including an ODD + D protocol [31], are included as supplementary materials.

### 1.2. MIDAS mechanisms

MIDAS applications simulate individual agents that share information and resources across dynamic social networks, and make boundedly rational, prospect theory-based choices e among ‘livelihood’ portfolios of utility streams—including income opportunities, resource sharing among social connections (e.g. remittances), or use values from places or assets (e.g. living in a home, enjoying nature). If the best portfolio of opportunities for an agent is in a different place, and the agent can afford to make the move, the agent migrates (supplementary figure S1; ODD + D protocol [31] in supplementary materials SI3). Agents share information and resources across their networks, sharing more with those they have stronger connections with. Connections are dynamic, fading over time but strengthened by interaction, and better enabled by sharing some of the same spaces or utility sources. Expectations on resource sharing are incorporated into agent decisions on livelihood portfolios, so that while decisions are made as individuals, they embed some elements of collective decision making. This operationalization avoids the conceptual challenges of using households as a basis for decision-making—that households may not tie closely to important social units [32], or that migrants participate in multiple ‘households’ simultaneously [33]—and follows the thinking that households as concepts to demarcate sharing across networks ought to be understood at best to be ‘fuzzy’ [34], with important connections across the extended family [35] and beyond. In each time step in a simulation, MIDAS loops through a newly randomized ordering of agents, allowing them to age (and possibly die), as well as to (with agent-specific probabilities): give birth, meet new agents, share information and interact, learn through observation, and reconsider their livelihoods portfolio. A process flow diagram for this algorithm is included as figure S18 in supplementary materials.

### 1.3. Application to Bangladesh and calibration over recent district-level migration

Livelihood portfolios in our MIDAS application include agricultural and non-agricultural income opportunities resolved to the district level using three waves of the Bangladesh Income and Expenditure Survey (2005, 2010, 2015). We calibrate our application to annual-average inter-district flows measured in the Bangladesh Sample Vital Registration Study (2002–2011) (supplementary table S1). We find our application to be sensitive to a handful (5) of the parameters included in our calibration space: access to credit; the number of locations considered in livelihoods decisions; risk tolerance; the degree of information sharing across agents; and the overall likelihood that an agent reconsiders their livelihoods in a timestep. We discuss these parameters in detail in section SI2.1.9. The model applied here explains approximately 26% of the variation in average inter-district migration over this period, compared to approximately 3% of variation in our null model (supplementary information SI2).

### 1.4. Projection under anticipated flooding

We applied the calibrated model to projections of future migration response to increased flooding events under different emissions scenarios [36]. The migration effects rely on the expected impacts on income sources under SLC to 2100 by coupling several models and datasets (supplementary table S1).

First, we simulate demographic change over this period using United Nations Department of Economic and Social Affairs estimates for fertility rates and World Health Organization estimates for mortality. Second, we estimate flooding with a statistical model for annual peak flooding depth out to 2100 (supplementary information SI2.2) under Representative Concentration Pathways (RCPs) 2.6, 4.5, and 8.5 [37]. Third, we translate flood depth into income damages via a published econometric relationship derived from a panel study examining fallout from the largest compound (pluvial and fluvial) flood event of the last century in Bangladesh (1998) [38]. Mechanistically, SLR-driven flooding will likely differ from pluvial and fluvial flooding in both impacts and perception; we use the 1998 flood as our best estimate of past flood damages and as a bounding box on expected SLR-flooding damages (see supplementary SI2 for discussion of this assumption).

Importantly, our flood damage relationship expresses damages in terms of the flood shock experienced, which is a relative measure capturing the additional flood depth above normal conditions (which for our experiment are the expected flood depths predicted for the reference period 2005–2015 over which we have income and migration data). As well, as we lack data on individual perceptions of how much flooding is currently considered normal, we conduct a Monte Carlo simulation of what absolute flooding depth agents consider ‘normal’—i.e. varying the minimum flooding depth above which damages to wages apply, from 0.5 through to 1.5 times the statistical mean or expected flooding depth—across simulations and report on the importance of this in our results

### 1.5. Analyzing model outputs

The nature of agents in MIDAS as computational objects with their own properties (viz. age, risk tolerance, wealth, training, social connection, etc) allows us to generate *in-silico* ‘life histories’ of agent utility portfolios—where they lived, how much wealth they derived, and how they did it—at three-month resolution. We use these output data to construct inter-district migration flows at quarterly resolution or above, and examine these flows to inform our first hypothesis. As well, we are able to tag agents who, within the subroutine to evaluate different utility portfolios and possibly choose something new, find themselves unable to afford any of the options that they are considering. Together with other details of their life histories (wealth, age, move history) and the broad pattern of inter-district migration, we use these tags to inform our second hypothesis.

Our second hypothesis makes a claim about the role of credit, and we outline here how it is defined in our MIDAS application. Agents can incur costs through moving, or by accessing new sources of utility (e.g. the cost of training to become a teacher, or the cost of purchasing farmland and machinery). We consider the cumulative spending by an agent to access new layers as a crude measure of capital, and assume that on average, agents with more capital are better able to access sources of credit. Lacking data on the absolute level of credit access in Bangladesh, our calibration exercise included a ‘credit access’ multiplier (varying from 0 to 2, uniform for all agents in the simulation) such that an agent’s access to credit was equal to this multiplier times their cumulative spending on accessing new utility layers (i.e. their ‘capital’). For further discussion of (i) model assumptions, and the robustness of model findings to these assumptions (supplementary information SI1); as well as (ii) calibration efforts, the five parameters shaping our calibration results, and the limitations of our calibration method, see supplementary information (figures S13, SI2.1.8 and SI2.1.9).

## 2. Results

**Figure 1.**
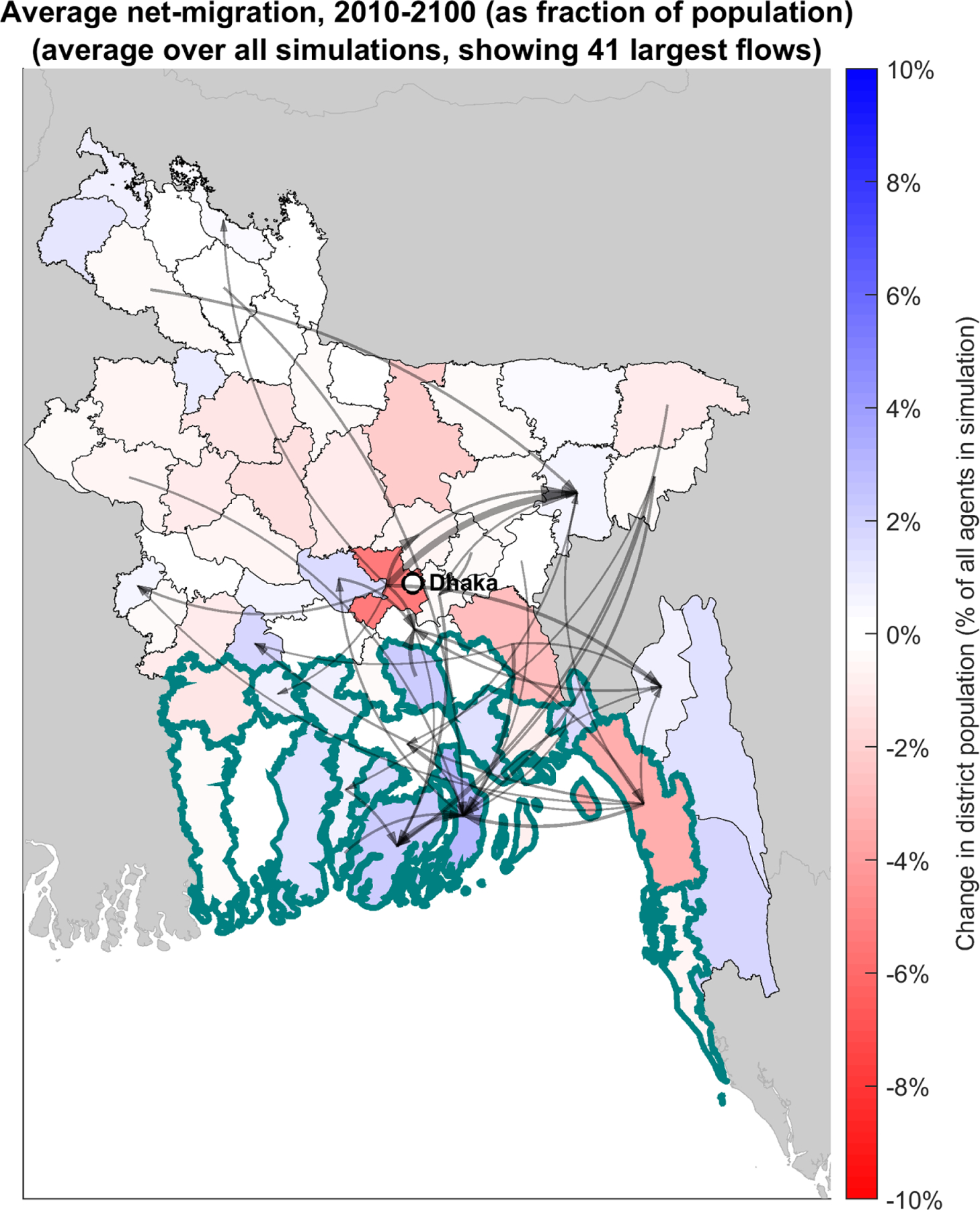
Projected net migration over the period 2010–2100, by district, across all simulation data. Net changes in migration are normalized by total agent population in simulation. Black arrows depict the largest 1% of all interdistrict flows, with thicker arrows indicating larger flows. 19 Coastal districts [40] highlighted with thick boundaries.

Under the three RCP scenarios considered, we find no amount of flooding from SLC causes enough damage to generate net out-migration from coastal Bangladesh after aggregating outcomes across 871 model runs [(with between 3 and 8000 migrants)] (figure 1; separate panels for each RCP scenario included as supplementary figure S6). Vulnerable coastal districts remain some of the top country-wide migration destinations under all emission scenarios, including RCP 8.5 that has the greatest SLC associated flood damages. A third of the 41 largest flows shown in figure 1 arrive in Bhola district, where a number of current establishments in retail and trade, as well as manufacturing employ 140 000 people [39]. SLC-related flooding drives a transition from agricultural income to non-agricultural income in coastal districts, and concentrates migrants (where there are urban opportunities) in coastal cities. At the same time, migration from the coast toward the interior is suppressed as the negative impact of flooding on wages accumulates, rendering labor migration less and less accessible. While stopping short of claiming that SLC-flooding will not drive widespread migration away from the coast, this finding allows us to falsify the hypothesis that it necessarily will. Specifically, the results suggest SLC-associated damages alone are unlikely to drive widespread migration away from coastal communities in Bangladesh.

**Figure 2.**
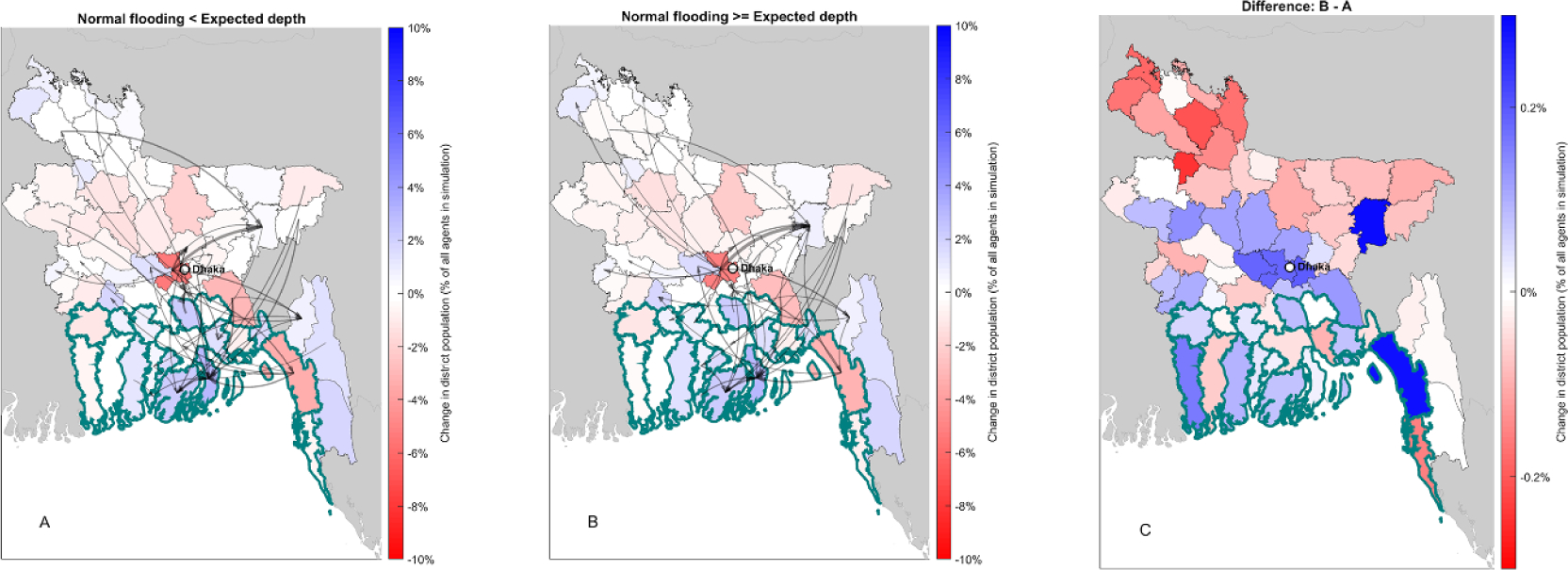
The effect of variation in agents’ experience with flooding on model outcomes: Panels depict average net-migration per district, 2010–2100, expressed as a fraction of total population, where flooding is (A) greater than expected, and (B) less than or equal to normal floods; and (C) the differences in net population between the two cases. Overall, experience with flooding drives coastal migration. Black arrows depict the largest 1% of all interdistrict flows, with thicker arrows indicating larger flows. 19 Coastal districts highlighted with thick boundaries.

Our results identify two important groups of agents: (a) those inclined to move to the coastal districts (for both agricultural and non-agricultural opportunities); and (b) people who are unable to find an affordable, improved alternative livelihood portfolio elsewhere, who we might describe as ‘immobilized.’ We employ a random-forest algorithm to identify the parameters that best explain differences in the size of these groups across our experiment (supplementary figure S3), with SLC flooding and its associated damages emerging as the most important parameter by far. Specifically, the greatest amount of variation in pro-coastal migration across simulations is explained by differences in the perceived ‘normal’ flooding depth—that is, the extent to which SLC flooding is experienced as a shock and draws damages in our model has the strongest influence on migration processes. We allow this to vary across simulations via a Monte Carlo draw from a uniform distribution from 0.5 through to 1.5 times the statistical expected flood depth (e.g. a drawing of 0.93 would lead all floods up to 0.93 times the expected flooding depth from our flood model to be experienced as normal, and flooding depths above this to draw damage via our damage model). The more that SLC flooding is experienced as shocks with damages, the less net migration towards the coast we observe, as expected. However, we still observe strong migration toward the coast under all degrees to which the simulated flood is experienced as a shock (figure 2).

**Figure 3.**
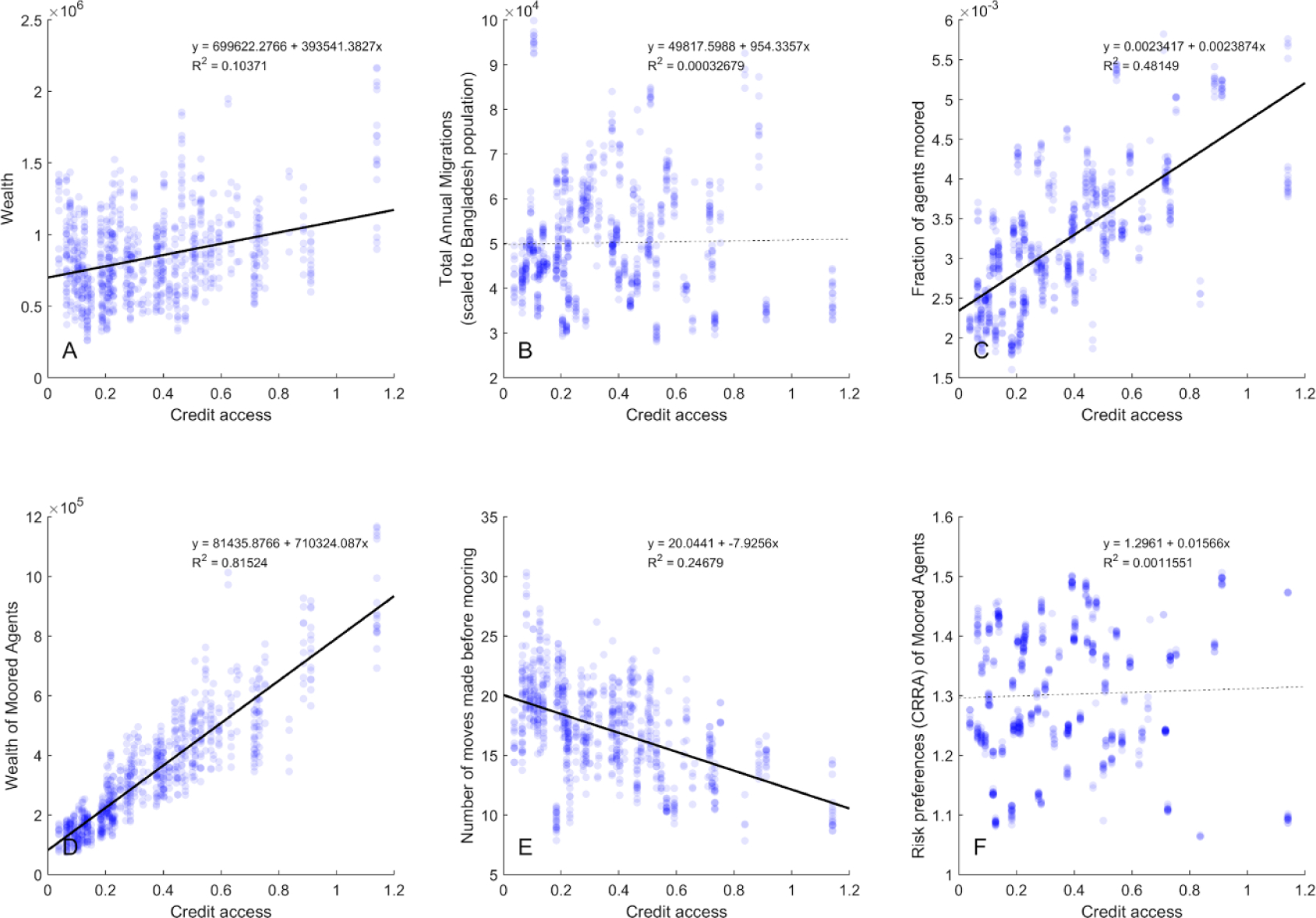
Top row panels show effects of increasing credit access on overall (A) agent average wealth, (B) total migrations, and (C) those agents unable to find better portfolios of opportunity than they currently have that they can afford. Bottom row panels show effects of increasing credit access on characteristics of those agents identified in panel (C), which we identify as moored: (D) their average wealth, (E) number of moves before becoming ‘moored’, and (F) their constant relative risk aversion (CRRA). Credit access is a scalar multiple of the amount an agent has spent to gain access to utility layers (analogous to investing in schools, training, or purchasing assets, e.g.), which we use as a proxy for capital. Significant trends with credit access are shown with solid black trendlines; non-significant trends are shown with dashed lines.

Access to credit is the greatest impediment for those who find it difficult to leave coastal districts, supporting the growing evidence that liquidity constraints inhibit mobility [14, 15]. increased access to credit leads to increased wealth on average (figure 3(A)), it does not increase mobility in general (figure 3(B)). Furthermore, it raises the share of agents who lack alternative, better options that they can afford (figure 3(C)). The lack of migrant opportunities reflects some interaction among (a) the dynamics of desirable labor markets being saturated with workers, (b) agents having fewer social contacts in places with better options to facilitate movement, and (c) increased agency to cater toward mooring preferences with wealth.

To gauge understanding of the drivers of this phenomenon, we explore what are the characteristics of these ‘immobilized’ agents in the context of rising access to credit. While they do not differ behaviorally by their risk preferences (figure 3(F)) or discount rates (not shown), they are wealthier (figure 3(D)), younger (not shown), and will have moved less frequently before this new state (figure 3(E)). These findings refute hypothesis 2. They also suggest that the language of ‘trapped’ populations in this context is less apt a description than ‘moored’—improved access to credit leads to investment in the livelihoods in place, more tightly mooring these agents to the set of livelihood choices they have made. This function of credit access to entrench people more tightly in place, against the broader trend of improved mobility, suggests the importance of ‘moored’ as well as ‘trapped’ in explaining why people stay in place.

The mechanisms we identify are spatially overlapping. Districts with the greatest net in-migration coincide with districts having the greatest percentage of ‘moored’ populations, with this relationship significantly stronger (more highly correlated) in coastal districts than in interior districts (supplementary figure S4). Livelihood opportunities in coastal areas continue to attract migrants, but when SLC-associated flood damages occur, households in vulnerable areas would be unable to afford to migrate out of the area. Much like many deltaic regions around the world [11], these results suggest that continued population growth in coastal Bangladesh is likely to be driven by continued in-migration, despite SLC associated damages. Many agents migrating to coastal districts subsequently find themselves or their descendants immobilized by the economic damages caused by SLC and the lack of available livelihood alternatives to migrate. These results are robust to assumptions on flooding expectations (figure 2) and credit access (supplementary figure S5).

## 3. Discussion

Our principal finding that flood impacts on income are not sufficient to drive out-migration from coastal districts by 2100 runs counter to common narratives about SLC and migration, and to some existing findings on distress migration from the coast [17, 41]. It provides a plausible, empirically calibrated quantitative account of the livelihood transitions and individual decision-making that could drive migration scenarios for LECZs, such as described in the UK government’s Foresight report on migration and global environmental change [12]. While this report predicted these isolated scenarios (aligning with 3 of the 4 scenarios in the report—A, B and C), our model outputs describe a future in which they occur simultaneously in the same locations. In our model, migration is accelerating toward coastal destinations (Scenario A), households are diversifying income to manage risk (Scenario B), and a portion of the population faces diminishing livelihood choices and migration alternatives as impacts accumulate (Scenario C). Beyond supporting these scenarios, our model also shows that when we increase access to credit, the effect may in some cases be to increase ‘mooring’, or stickiness to place. This provides an additional perspective within immobility beyond ‘trapped’ as it implies a sense of agency amongst vulnerable populations. While a natural conclusion from our findings would be to encourage policies that promote credit access, our model projections suggest it is unlikely that broadening access to credit will alone solve mobility problems.

In a context where agricultural activities can be easily adapted but there are significant land market frictions (i.e. access to land is not so open), our model provides a plausible counter-narrative to the strong literature expectation for livelihood transitions under projected SLR scenarios, and identifying the specific mechanisms that drive these model outcomes, namely access to credit and experience of flooding. Access to credit reinforces the role of credit in poverty reduction and speaks to the impact that policy responses to hazards can have on mobility and vulnerability. Future migration modeling exercises could thus be improved by including assumptions on potential policy responses, including the provision of grants to buffer enterprises against natural disasters [42, 43] or programs promoting precautionary savings behavior [44], and examine the appropriate timing or conditions to trigger those responses [45]. As shown here, the nature of how credit is delivered and received is likely crucial to invoke particular behavioral responses as we discovered in our findings. When credit access is increased via a pure income shock, many agents in the model decide to stay in flooded areas. Likewise, future migration models should prioritize simulating how other policy interventions, such as regulations on zoning, housing construction, or reforestation, can alter experience with hazards and thus migration tendencies.

Our results provide important new understanding of climate change adaptation research by constructing future migration scenarios from the bottom up, based on the emergent outcomes from many individual, heterogeneous migration decisions. However, improving these scenarios requires better knowledge on, and quantification of, human behavior and social mechanisms. First, we have drawn attention to the need to better understand how to draw on historical, empirical analogs for flooding events and associated shocks (e.g. to income) to make realistic estimates about the future. Second, we have highlighted the need for better quantitative data on how people adapt to shocks, and how quickly the unexpected can become the norm. Finally, since we cannot experiment on vulnerable populations, we need models that provide realistic expectations about policy responses and how they might influence access to credit and livelihood opportunities, both now and dynamically in the future. While we can speak qualitatively of plausible future pathways, our capacity for prediction is limited without better efforts at assembling relevant data particularly on thresholds of exposure, and interpretations of risk [6], as well as implications of loss and damage [46]. Future models with policy-actionable outputs will be served with information observed in flood survivors, those living with floods, and decision-makers learning from disasters, which can inform progressive policy responses to SLC [4]. Importantly, our efforts in the present study are a *ceteris paribus* examination of the effects of flooding on individuals via damages to income streams, holding the broader macro-economy constant. Should coastal risk in a future Bangladesh lead the economic sectors that are driving current coastal opportunities to relocate elsewhere, our findings would likely differ (see discussion in SI1.3). Alongside the need for better datasets is the need to couple models of individual decisions to models of the macro-economy (e.g. computable general equilibrium, or CGE, models), for which we have recently seen promising proofs of concept [47]. In addition to simulating individual income decisions, future models should incorporate economic planning scenarios.

The dominant narrative is that inundation from SLC will lead to large-scale displacement and migration from coastal areas. However, our results show that the opposite is as well plausible for Bangladesh: the economic amenities of coastal areas could lead to continued in-migration despite SLC flooding. Further, our results show that while the accumulated damage of SLC to livelihoods may eventually undermine people’s ability to move away, immobilizing many coastal in-migrants in increasingly hazardous coastal areas, there are other factors behind staying in place that need to be disentangled. Migration, when it takes place, is associated with experience of flooding, showing that people have their own thresholds to risk and damage that are eventually exceeded. Thus, simple inundation-migration models, by failing to incorporate the livelihood context and behavioral responses, miss these important dynamics. Rather than SLC creating out-migration, our results show how livelihood diversification and access to credit could plausibly help a growing coastal population continue to adapt against its negative impacts on agricultural productivity.

## Supplementary Material

Supp Materials

## Data Availability

The model simulation outputs generated during and analysed during the current study are available from the corresponding author on reasonable request. The income, expenditure, and migration data that support the findings of this study (table S1) are available from the Bangladesh Bureau of Statistics, but restrictions apply to the availability of these data, which were used under license for the current study, and so are not publicly available. All other data generated or analysed during this study are included in this published article (and its supplementary information files). The data that support the findings of this study are openly available at the following URL/DOI: https://doi.org/10.5281/zenodo.3774666. All MIDAS inputs, code, and analysis scripts used in this study are available at 10.5281/zenodo.3774666.
