# Supplementary material for: Migration towards Bangladesh coastlines projected to increase with sea-level rise through 2100
Source: Environ Res Lett. Author manuscript; Available in PMC 2022 Aug 26. (PMC9415774; doi:10.1088/1748-9326/abdc5b)
Supplement: Supp Materials [file NIHMS1777602-supplement-Supp_Materials.pdf]

Supplementary Information

This document contains 3 parts. Section SI1 includes a brief overview of MIDAS principles, sensitivity analysis, and discussion of key modeling assumptions, along with additional tables and figures referenced directly in the article text. Section SI2 provides a detailed description of details specific to this application of MIDAS to Bangladesh, details of our calibration efforts, and discussion of the limitations of this calibration. Finally, Section SI3 reproduces the ODD+D (Overview, Design, and Details+Decisions) document for the general MIDAS modeling framework.

SI1.1 - MIDAS Summary

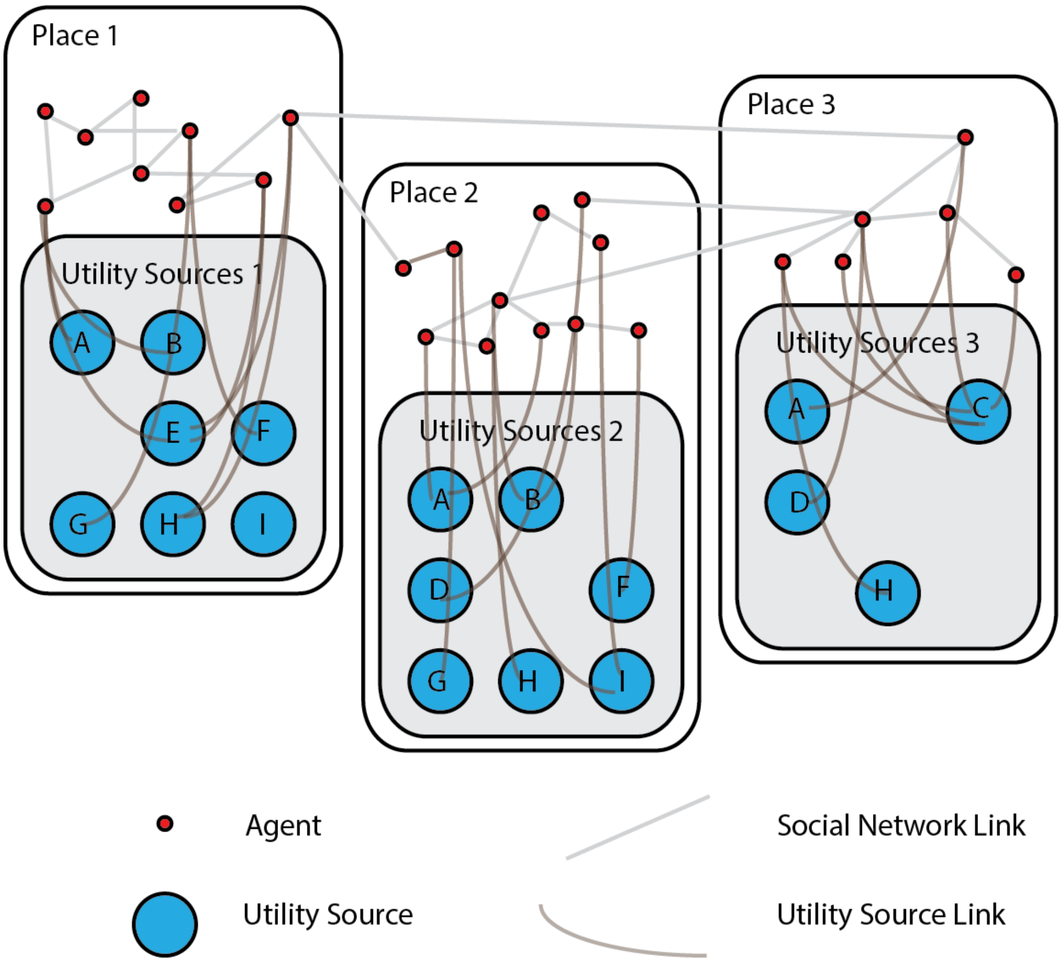

Fig M1 | Basic agent based model design: Agents (red dots) were programmed to realistically simulate individual livelihood decision-making among a set of income alternatives (blue dots), social networks to convey information and opportunities (straight gray lines) in an array of locations.

The purpose of the MIDAS framework is to develop model simulations of livelihoods decision making at the individual and household level, including intensification (focus on a small number of activities for income), diversification (spreading across a larger number of activities for income), and migration (changing locations to access opportunities for income), as functions of available opportunities varying in space and time. MIDAS models individual agents deriving livelihoods, connected to each other by social networks, and existing at particular ‘places’ – points in a two-dimensional space (Figure M1).

Individual agents make decisions on the best portfolio of utility-yielding activities (including income sources, assets, as well as any other feature from which an agent may derive utility over time) across a sample of different places, including their current place and current portfolio. A complete description of the MIDAS framework following the ODD+D Protocol(Karlan, Ratan, and Zinman 2014; Müller et al. 2013) is included in this document as Section SI3, while a full description of calibration activities is included as section SI2.

The decision model used in the current MIDAS version (Fig. S1) embeds the following assumptions with roots in the decision literature:

- Factors shaping livelihoods decisions (of which migration is one) are well described by 'pushes' (declines in opportunity local to the agent), 'pulls' (availability of opportunities distant from the agent), and 'moorings' (investments and ties – such as assets o(Moon 1995; Stimson and McCrea 2004)r family – that cannot be easily moved with the agent)(Moon 1995; Stimson and McCrea 2004).
- Decisions regarding portfolios of livelihoods opportunities are boundedly rational, such that only a small number of possible competing opportunities are considered at one time(Rubenstein 1998; Kahneman 2003).
- Given two income streams of equal nominal value, a risk-averse agent should prefer the less time-varying of the two, such that a livelihoods portfolio that spreads activities across activities with uncorrelated risks (diversification) can be a risk minimization strategy; this can be accomplished via an expected utility framework(Feder 1980; Bert et al. 2011; Hong 2015) based on constant relative risk aversion(Dave et al. 2010).
- In considering future income streams, agents will dislike losses approximately twice as strongly as they enjoy equivalent gains, as in prospect theory(Kahneman 2003; Schlüter et al. 2017).

These assumptions allow that: i) migration can emerge as an adaptive strategy alongside other livelihoods strategies (such as diversification or intensification) without being hard-coded in (e.g., as a stage-wise decision), ii) preferences for crop diversification along a season, or commuting/local migration over short periods, can emerge in the same modeling space, and iii) factors outside of income, such as assets or preferences for family connection, can shape livelihoods decision-making (conditional on the availability of data on relative preferences). Agents age over time, and MIDAS allows for agent age to affect a range of agent parameters (such as time horizon for decision making) via scripts specific to an implementation of MIDAS. Agents may die or have children, following age-specific mortality and fertility data. Additionally, agent social networks decay over time, and will weaken unless they are enhanced again by interactions or the sharing of remittances. Agents interact directly via social interactions that include the exchange of knowledge of past utility, and by sharing their received utility across their social networks.

Our application of MIDAS to Bangladesh is described in detail in the Supplementary Material for this study. We model demography (age, gender, and location) at simulation start based on age- and gender-disaggregated district-level population data from the 2011 Bangladesh Population Census, made available via IPUMS International (Minnesota Population Center 2015). We model mortality and fertility with age- and gender-disaggregated data from the Global Health Observatory data repository of the WHO (WHO 2015), and the World Fertility Data 2012 dataset produced by the United Nations Department of Economic and Social Affairs (UNDESA 2013). We derived all utility layers for this

simulation (including agricultural and non-agricultural income) from three waves (2005, 2010, 2015) of the Bangladesh Household Income and Expenditure Survey (HIES).

We applied an Approximate Bayesian Calibration (ABC) approach over 65 model parameters of varying scope (Supplementary Table S12), calibrating against the relative inter-district migration rate Q, averaged across the years 2002-2011 using data from the Bangladesh Sample Vital Registration System, with flows weighted by the populations of both source and destination districts, and achieving a weighted Pearson Correlation of 0.2561 across all interdistrict flows. We then projected migration flows using exceedance maps of annual peak flooding for Bangladesh at decadal intervals out to 2100 (likelihoods of annual flooding exceeding depths of 0.1m to 5m in increments of 0.1m; Supplementary Material SI2.2) to instantiate plausible annual peak floods for Bangladesh, and translated those floods into economic damages using a damage function for flood depth on agricultural and non-agricultural income derived from a previous study (Mueller and Quisumbing 2011). Subject to availability of shared high-performance computing facilities, we completed a total of 871 simulation runs across RCP 2.6 (255 runs), RCP 4.5 (294 runs), and RCP 8.5 (322 runs). Across these 871 runs, we varied the degree to which flooding was experienced as a 'shock,' subject to damages as defined by our damage function, at the simulation run level uniformly from 0.5 through to 1.5 times the statistical expected flood depth.

SI1.2 Sensitivity Analysis

Variation in model parameterization across the ensemble of high-performing calibrations, together with the Monte Carlo experiment on the degree to which flooding is experienced as a shock, provide a rich dataset for sensitivity analysis. Relative importance of parameters in shaping migration outcomes was estimated using Matlab's treebagger algorithm using forests of 1000 randomly generated regression trees; parameter importance is estimated by the relative increase in prediction error when that parameter is excluded from the generation of trees, compared to the error rate when it is included in the generation of trees. Figures 2 and S3 show the top 10 most influential model parameters in shaping migration outcomes in our simulations, as measured by the largest relative prediction errors arising from their exclusion from tree generation.

SI1.3 Implications of model assumptions for key findings

Models need not include all things, but model results should be robust to the relaxation of critical simplifying assumptions. We were able to proceed with existing datasets by i) treating both migration and flooding at the district scale; ii) using a historical analog for flooding to imagine damages from future sea level change; and iii) allowing the Bangladesh economy of the future to look like the economy of today. Our principal finding – that anticipated flooding under SLR forcing is not sufficient to suppress or reverse migration toward coastal districts – has to be examined in the context of these three critical assumptions.

Firstly, our treatment of migration at the district scale may be evaluated directly from the results of our calibration – we explain about 26% of inter-district migration with our model, compared to 3% with a null model. We do not have a similar means of calibrating flood response. Real floods are not uniform within district boundaries, and heterogeneous flooding within districts might lead to different patterns of intra- and inter-district moves that we don't capture. Our district-level treatment could be biased if higher flood risks [impacts/damages?] were systematically correlated with higher wage levels at a sub-district level, as we may end up underestimating the damage to these strong pulls from flooding when we average flood expectations across the district. However, we observe no such association in our data (Figure S2) and are satisfied that our finding would be qualitatively robust to treatment at higher resolution.

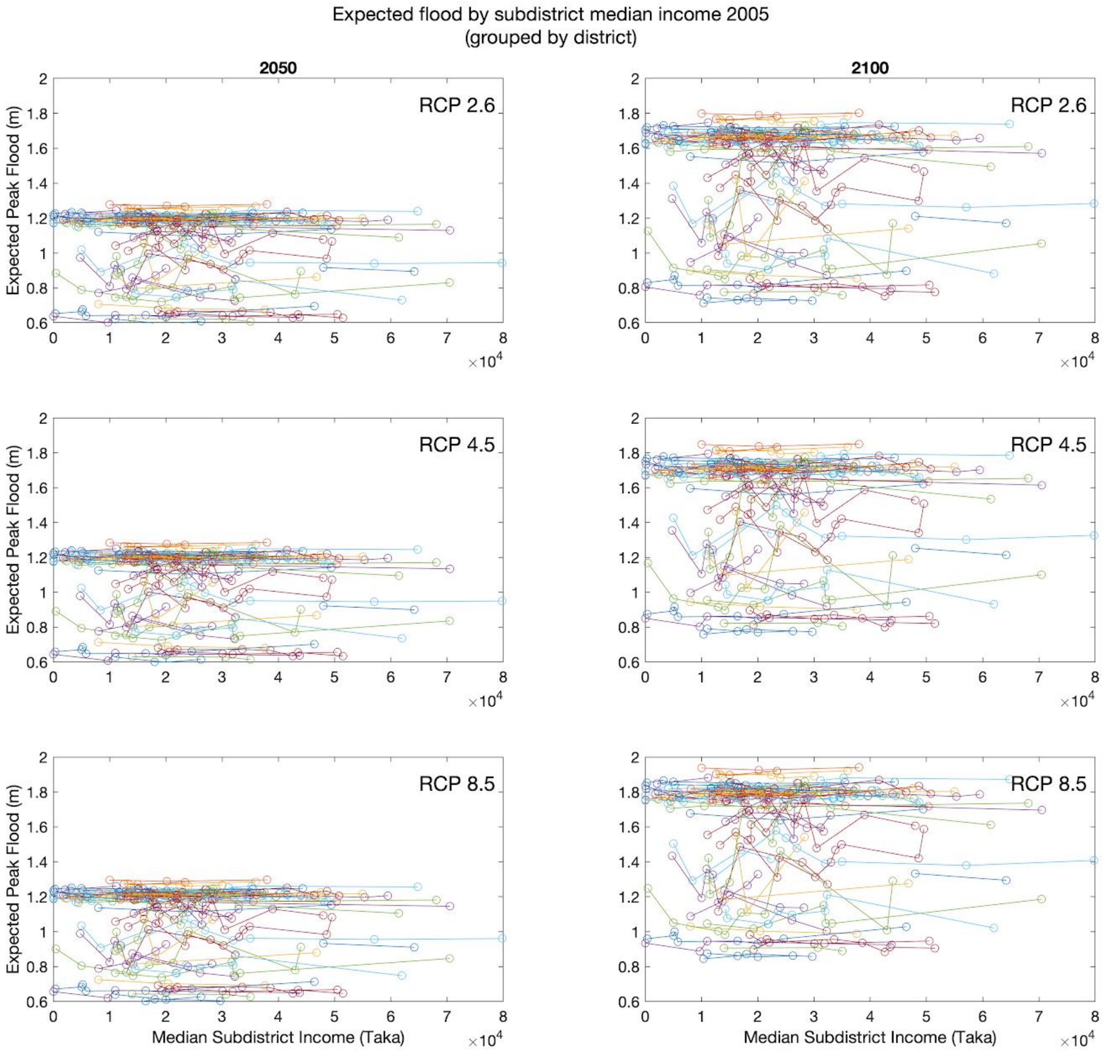

Fig S2 | Expected peak flood depth as a function of median subdistrict income (circles), grouped by district membership (colored lines), across all RCPs for periods 2050 and 2100. Income is not a significant predictor of expected peak flood depth within districts (OLS regression of flood depth on median income, controlling for districts as covariates) in any of the six panels shown.

Secondly, the 1998 flood we used to estimate a damage function for flooding is mechanistically different from flooding due to sea level change. However, it is the most extensive and severe flood for which data are available, and represents flooding largely experienced as a shock, above the baseline of flooding that is experienced as a norm. The processes leading to increased SLR flooding are incremental over time, so that we would not expect SLR floods of comparable depth to be experienced entirely as shocks in the way that the 1998 flood was. We expect then that our damage model is reasonably an upper bound on depth-specific damages; floods under SLR are more likely to be expected, and experienced less as a shock that was not in some way prepared for. They may reasonably be expected to cause lower damages, and be less likely to discourage migration flows toward the coast than our analyses have

found. Furthermore, our sensitivity analysis over the range of 'flood experienced as shock' gives us confidence that our finding would be robust to improvements in this damage model.

Lastly, our analysis should be considered a *ceteris paribus* examination of the effects of flooding shocks, with much of the broader socio-economy held constant. It would be entirely fair to claim that should the macroeconomy of a future Bangladesh be broadly different than today, with favorable economic opportunities in areas distant from flooding, our finding would be different. However, we do not have the datasets or mechanistic understanding to project forward what shape Bangladesh's economy could take decades from now; further, toolkits for coupling macroeconomic movement to individual-level decisions (e.g., ABM and computable general equilibrium (CGE) models) are at best in proof-of-concept stages (e.g. Niamir et al. 2018). Together with the other two assumptions above, this highlights the challenge of building out datasets for meaningful future projections under SLR over data-poor environments like Bangladesh.

Table S1: Data Sources

Variable	Source	Description	Units	Data years
Demographic distribution	Bangladesh Population Census	District level population by age group (5-year intervals) and gender	Persons/category	2011
Mortality	Global Health Observatory	Mortality rates disaggregated by age group (5-year intervals) and gender	Death rate (deaths per 100 people) /year/category	2015
Fertility	World Fertility Data	Fertility rates disaggregated by age group (5-year intervals between 15 and 50) for female persons	Birth rate (births per 100 people) /year/category	2012
Income	Bangladesh Household Income and Expenditure Survey	Household-level microdata on income from non-agricultural and agricultural sources	Taka/year /household /category	2005, 2010, 2015
Migration	Bangladesh Sample Vital Registration System	Individual-level microdata on in- and out-migration and demographic characteristics, nationally representative at district level	Persons	2002-2011
Flood projections		High spatial-resolution maps of the likelihood of flood exceeding depth X at least once in a year, for X spanning 1-5m in 0.1m intervals	Probability	2000-2100
Flood impacts	Bangladesh Flood Impact Panel Survey	Estimation of relative reduction in income per foot of flood shock for non-agricultural and agricultural income sources in both the year of, and year following, a flood shock, based on Mueller and Quisumbing (2011) analysis	% change in wage/foot	1998-2004

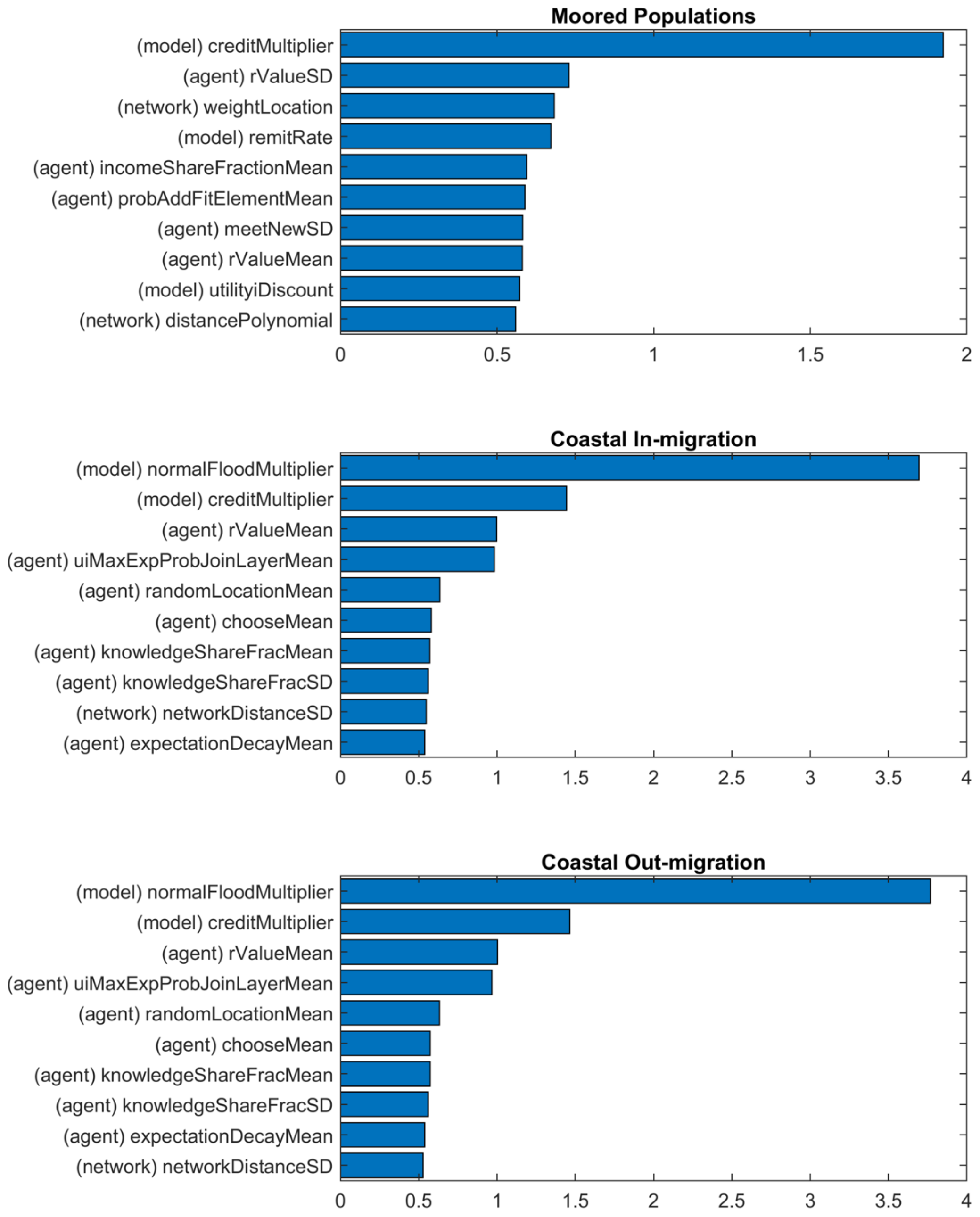

Fig S3 | The mechanisms underlying the emergence of 'moored' populations, and rates of coastal in- and out-migration. Panels depict the relative explanatory importance of top 10 key parameters (out of 70 calibration parameters overall) in simulating these outcomes across our experiments. This measure is expressed as the relative increase in prediction error between predictions from a forest of 5000 regression trees built from a pool of all predictor variables, and a second forest built from a pool that excludes the predictor of interest. By a wide margin, the parameter 'creditMultiplier' (a simulation-wide parameter scaling the level of access to credit for agents) is central to shaping moored populations; and the parameter 'normalFloodMultiplier' (a simulation-wide parameter scaling the degree to which flooding across the simulated space is experienced as normal (as opposed

to as a shock)) is central to shaping coastal in and out migration. Full list of calibration parameters and brief description in Supplemental Table S12.

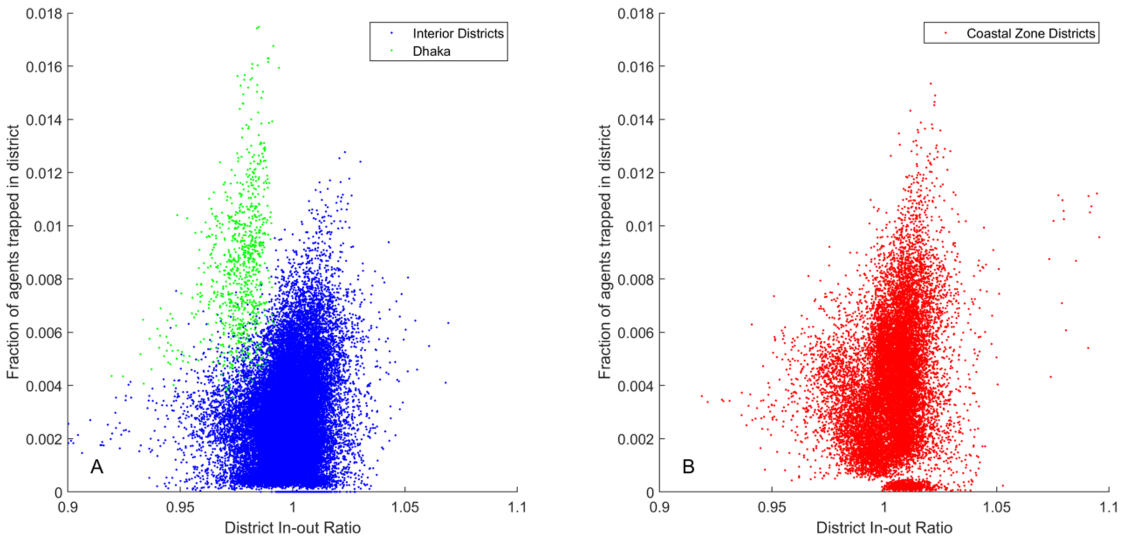

Fig S4 | Relationship at the district level between net migration (expressed as the in-out ratio, i.e., values greater than 1 indicate net in-migration) and 'trapped' agents (i.e. agents can find no affordable alternative livelihood portfolios to switch to, in the same district or otherwise) for A) interior and B) coastal zone districts. Each dot represents one district in one of the 871 simulation runs. Coupling of trapped fraction and in-out ratio is stronger for coastal districts than interior districts, measured via dummy regression: $Y = -0.0142 + 0.0171X + 0.0011DX$, where Y is the fraction of trapped agents, X is the in-out ratio, and D is a dummy variable equal to 1 for coastal districts and 0 otherwise. All coefficients are significant at 99%, indicating that trapped populations are tied more to in-migration in the coastal zone than in the interior. Metropolitan district of Dhaka shown separately in green in (A), with relatively high fractions of trapped agents across most simulations even with net out-migration (in-out ratio below 1), reflecting Dhaka's function as a nexus from which some migrants lose the capacity to leave.

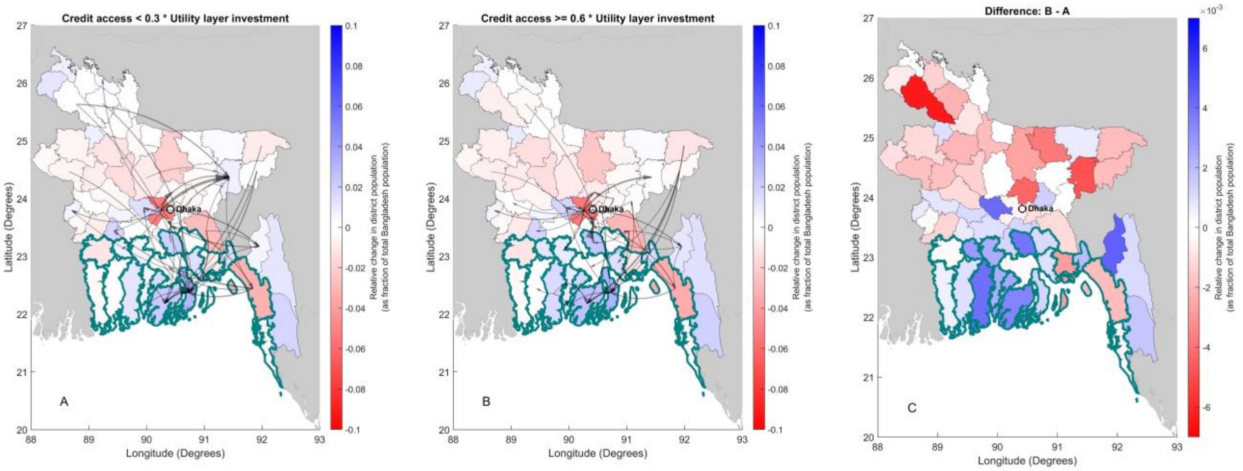

Fig S5 | The effect of variation in agents' access to credit on model outcomes: Panels depict average net-migration per district, 2010-2100, expressed as a fraction of total population, where access to credit is A) low, and B) high; and C) the differences in net population between the two cases. Black arrows depict the largest 1% of all interdistrict flows, with thicker arrows indicating larger flows. 19 Coastal districts highlighted with thick boundaries.

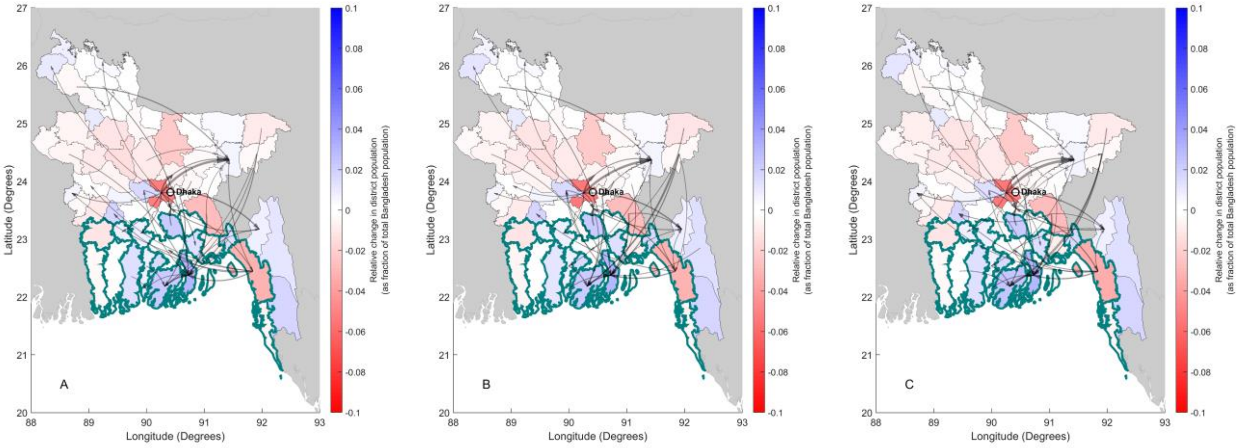

Fig S6 | The effect of carbon emissions on model outcomes: Panels depict average net-migration per district, 2010-2100, expressed as a fraction of total population, under emission scenario A) RCP2.6, B) RCP4.5, and C) RCP8.5. Black arrows depict the largest 1% of all interdistrict flows, with thicker arrows indicating larger flows. 19 Coastal districts highlighted with thick boundaries. We do not observe major differences across the three RCP scenarios, nor do dummy variables for the RCPs appear as important in our random forest test of variable importance (Figs. 2, S2)

Literature Cited

Bert, Federico E., Guillermo P. Podestá, Santiago L. Rovere, Ángel N. Menéndez, Michael North, Eric Tatara, Carlos E. Laciana, Elke Weber, and Fernando Ruiz Toranzo. 2011. "An Agent Based Model to Simulate Structural and Land Use Changes in Agricultural Systems of the Argentine Pampas." *Ecological Modelling*. https://doi.org/10.1016/j.ecolmodel.2011.08.007.

Dave, Chetan, Catherine Eckel, Cathleen Johnson, and Christian Rojas. 2010. "Eliciting Risk Preferences: When Is Simple Better ?" *Journal of Risk and Uncertainty* 41 (3): 219–43.

Feder, G. 1980. "Farm Size , Risk Aversion and the Adoption of New Technology under Uncertainty." *Oxford Economics Papers* 32 (2): 263–83.

Hong, Gihoon. 2015. "Examining the Role of Amenities in Migration Decisions: A Structural Estimation Approach." *Papers in Regional Science: The Journal of the Regional Science Association International* 95 (4). https://doi.org/10.1111/pirs.12154.

Kahneman, Daniel. 2003. "Maps of Bounded Rationality: Psychology for Behavioral Economics." *The American Economic Review* 93 (5): 1449–75.

Karlan, Dean, Aishwarya Lakshmi Ratan, and Jonathan Zinman. 2014. "SAVINGS BY AND FOR THE POOR: A RESEARCH REVIEW AND AGENDA." *Review of Income and Wealth* 60 (1): 36–78.

Moon, B. 1995. "Paradigms in Migration Research: Exploring 'Moorings' as a Schema." *Progress in Human Geography* 19 (4): 504–24.

Mueller, Valerie, and Agnes Quisumbing. 2011. "How Resilient Are Labour Markets to Natural Disasters? The Case of the 1998 Bangladesh Flood." *Journal of Development Studies*. https://doi.org/10.1080/00220388.2011.579113.

Müller, Birgit, Friedrich Bohn, Gunnar Dreßler, Jürgen Groeneveld, Christian Klassert, Romina Martin, Maja Schlüter, Jule Schulze, Hanna Weise, and Nina Schwarz. 2013. "Describing Human Decisions in Agent-Based Models - ODD+D, an Extension of the ODD Protocol." *Environmental Modelling and Software* 48: 37–48.

Niamir, Leila, Olga Ivanova, Tatiana Filatova, and Alexey Voinov. 2018. "Tracing Macroeconomic Impacts of Individual Behavioral Changes through Model Integration." *IFAC-PapersOnLine*. https://doi.org/10.1016/j.ifacol.2018.06.217.

Rubenstein, Ariel. 1998. *Modeling Bounded Rationality*. Edited by K. G. Persson. Vol. 65. Cambridge, MA: MIT University Press.

Schlüter, Maja, Andres Baeza, Gunnar Dressler, Karin Frank, Jürgen Groeneveld, Wander Jager, Marco A. Janssen, et al. 2017. "A Framework for Mapping and Comparing Behavioural Theories in Models of Social-Ecological Systems." *Ecological Economics: The Journal of the International Society for Ecological Economics* 131: 21–35.

Stimson, Robert J., and Rod McCrea. 2004. "A Push – Pull Framework for Modelling the Relocation of Retirees to a Retirement Village: The Australian Experience." *Environment and Planning A: Economy and Space*. https://doi.org/10.1068/a36206.

Part SI2 - MIDAS Bangladesh Application Details

This document describes two applications of the MIDAS framework: 1) a calibration of MIDAS to district-level internal migration in Bangladesh, and 2) an application of the calibrated Bangladesh model to project migration flows up to 2100 under additional flooding driven by anticipated sea-level rise, and an estimation of these flood impacts on agricultural and non-agricultural income sources.

Section SI2.1 provides additional details on application-specific submodels not treated in the MIDAS ODD+D document, and describes the calibration procedure and result. Section 2 introduces the additional datasets and submodels used for the forward projections to 2100, and describes the experimental design used to produce our set of simulations.

SI2.**1 Calibration to Bangladesh inter-district migration**

This application simulates the period from 2005 to 2015 as 44 quarterly annual timesteps with an additional (randomized) number of timesteps added to the beginning of the simulation as spin-up time. This document supplements the main MIDAS ODD documentation, describing data and submodels specific to this application and not general to MIDAS. Specifically, it summarizes our approach to estimating the following model inputs:

1. **Initial demographic distribution**
2. **Mortality and fertility**
3. **Additional modeled effects of demography**
4. **Social network structure**
5. **Utility layers**
6. **Moving costs**
7. **Remittance costs**

SI2.***1.1 Initial demographic distribution***

We model demography (age, gender, and location) at simulation start based on age- and gender-disaggregated district-level population data from the 2011 Bangladesh Population Census, made available via IPUMS International (Minnesota Population Center 2015). Specifically, for each agent initialized in a simulation, we assigned their location probabilistically based on census populations by district, then assigned gender probabilistically based on gender-disaggregated census populations within the assigned district, then finally assigned age probabilistically based on age-disaggregated census population within assigned gender and district. Age classification included the groups of 0 years, 1-4 years, 5-9 years and all 5-year groups up to 84, and a final group of 85-100 years.

SI2.***1.2 Mortality and Fertility***

Mortality data was taken from the Global Health Observatory data repository of the WHO (WHO 2015), with age- and gender-disaggregated mortality rates for age classes matching those of the Population Census data used for demographic distribution. Mortality rates are the same in all districts.

Fertility data was taken from the World Fertility Data 2012 dataset produced by the United Nations Department of Economic and Social Affairs (UNDESA 2013). Fertility rates are reported for females in age classes from 15-19 and all 5-year groups up to 49.

SI2.***1.3 Additional modeled effects of demography***

There is considerable flexibility in MIDAS to implement demography-specific effects on decision, structure, etc. In the current simulation, only one age-specific effect is implemented – a gradual increase in discount rate over time, such that older agents will discount the future more highly (and thus value possible returns from a change in portfolio less highly against the upfront costs). Agents' individual discount rates are randomly distributed with distribution parameters included as calibration parameters; however, the factor by which their individual discount rates change over time is not. It is presently a fixed data input (Figure S6), but there is room to explore this and other demographic effects on decisions and behavior in model extensions.

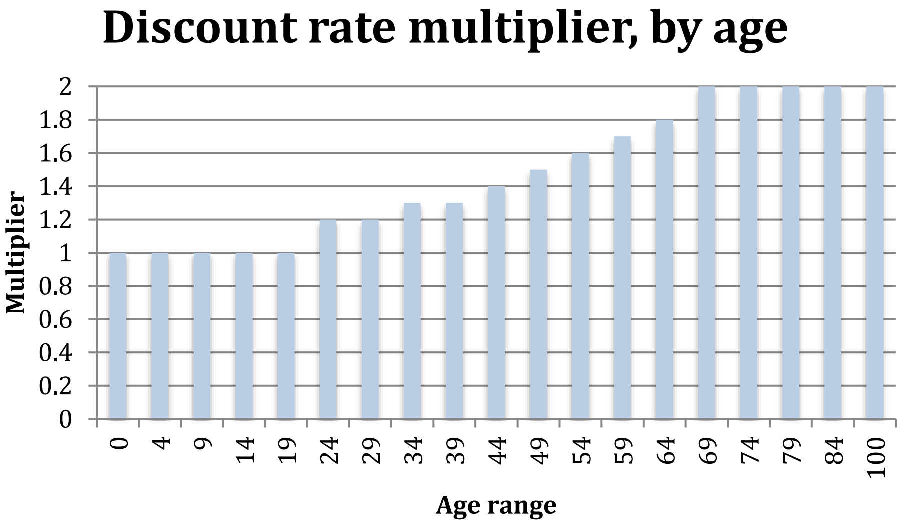

Figure S6: Discount rate multiplier

SI2.***1.4 Social Network Structure***

Social network connections in MIDAS are non-directed links between two agents *i* and *j*, described by a strength between 0 and 1 that influences the degree and likelihood of information and resource sharing in a simulation (see main ODD for more detail). Social network connections decay over time and are strengthened through interaction, via parameters set during calibration.

The network of connections among agents is constructed following a simple model. The number of network connections *n* that an agent *i* 'initiates' is drawn from the normal distribution $N(\mu_n, \sigma_n)$, where both μ_n and σ_n are calibration parameters. This is repeated for all *i*, generating a list of network connections for which one end of the link is defined. The ordering of this list is randomized, and then for each link, an agent *j* is selected probabilistically to be the other end of the link (note – links are not directed in MIDAS). The model used for identifying the likelihood of selection of an agent used in this application develops a weighted score of three factors: shared network connections, physical distance, and shared layers. The shared network connection score is the sum of the strengths of all links agent *i* has with other agents also connected to agent *j*, normalized by the highest raw connection score across all agents *j*. The distance score is calculated from the distance between agent *i* and agent *j*, raised to a polynomial (chosen by calibration) and normalized by all distances to agents *j*, such that shorter distances have nonlinearly higher scores. The shared layer score is estimated by the number of layers in the same place that agent *i* and agent *j* both occupy, again normalized by the highest number of shared layers between agent *i* and any agent *j*. The weights allocated to each of these three scores are also found by calibration.

SI2.***1.5 Utility Layers***

We derived all utility layers for this simulation from three waves (2005, 2010, 2015) of the Bangladesh Household Income and Expenditure Survey (HIES). Utility in timesteps representing the years 2005 to 2015 are developed as described below; spin-up time periods are a repeating cycle of the year 2005.

A complete specification for a utility layer includes i) the utility stream over time, ii) the time commitment of the layer as a fraction of total available time, iii) a classification of the number of openings in the layer as 'fixed'

(meaning only a certain number of agents may access the layer – e.g., a limited number of teaching jobs in a district) or ‘open’ (meaning no restriction on access, though the value of the layer may decline with excess occupation – e.g., too many taxi drivers competing for fares drives down individual earnings) and iv) a cost for initially accessing the layer, which may allow an agent to access the same layer in other places (e.g., passing a certification valid anywhere in a state or country) or only in one place (e.g., buying an immovable asset like a house). We describe below our identification of the set of included income sources as utility layers, and the estimation of these three properties of the included utility layers.

SI2.*1.5.1 Included layers*
We identified all income layers recorded in the survey as:

1. Remittance income
2. Transfer income
3. Rest of other income (including rental income)
4. Annual wage income
5. Annual in-kind income
6. Annual salary income
7. Sugarcane income
8. Jute income
9. Wheat income
10. Boro rice income
11. Aman rice income
12. Aus rice income
13. Oilseed income
14. Pulse income
15. Maize income
16. Income from other crops
17. Total livestock sales
18. Total fish sales
19. Total tree sales

We summed all income sources at the household level, and classified households into four income quartiles (Q4 highest, Q1 lowest).

Next, we selected income layers for inclusion in our model – we chose to exclude income sources 1, 2, and 5 (remittance, transfer, and in-kind) as those sources would be treated through social network interactions in MIDAS, making these layers more appropriate as possible calibration data; additionally, we excluded income sources 17 through 19 (livestock, fish, and tree sales) as at least one of these streams (livestock) appeared to have a larger order of magnitude than other sources, suggesting that they may not be directly interpretable as income (and rather, as revenue).

SI2.*1.5.2 Utility Stream*

We use the following formula for utility or 'value' for layer *i* in a particular place *j* at time *t*:

$$V_{ijt} = V_{base,ijt} \cdot \frac{m \cdot n_{expected,ij}}{\left(max\left(0, n_{actual,ij} - m \cdot n_{expected,ij}\right) \cdot k + m \cdot n_{expected,ij}\right)} \quad (1)$$

where $V_{base,ijt}$ is a base rate for the utility value estimated from reported income, as described below; $n_{expected,ij}$ is the expected number of agents occupying the layer, obtained by scaling the number of observed occupants of a layer in the HIES by n_{agents} / n_{HIES}, the ratio of the number of agents in the simulation to number of people in the HIES sample; $n_{actual,ij}$ is the number of agents actually occupying the layer; and *m* and *k* are parameters that shape how utility declines as the number of actual occupants exceeds the number of expected occupants. All agents occupying layer *i* in a particular place *j* at time *t* derive the same value from the stream, but have agent-specific coefficients specifying their utility on a unit of value from a particular source.

To develop the values $V_{base,ijt}$ for the included layers, we used our initial classification of households into income what do Q1 households earn on average in sugarcane income in Bogra?). Our dataset included values for each of the years 2005, 2010, and 2015 for each of 13 included income sources, each broken into four quartile layers, for each of 64 districts. We used Matlab's *interp* function to estimate the interior annual averages 2006-2009, 2011-2014.

At this point we have an array of 52 layers x 64 districts x 11 years. However, this application of MIDAS uses a quarterly annual time periods (i.e., four time periods per annual cycle, and 44 time periods in 11 years), so that annual income must be attributed to one or more of these steps. We assumed income sources 3, 4, 6, and 16 to be spread equally across all 4 quarterly periods (P), and consulted an agricultural calendar for Bangladesh (BBS 2017) for approximate harvest periods for crop income, such that the fraction of annual income earned in each quarter, for each layer, is as follows:

Table S7: Annual income spread across quarterly periods, by income source

	Rest of other income	Wage income	Salary income	Sugarcane income	Jute income	Wheat income	Boro rice income	Aman rice income	Aus rice income	Oilseed income	Pulse income	Maize income	Income from other crops
P1	0.25	0.25	0.25	1	0	1	0	0	0	0.25	1	0	0.25
P2	0.25	0.25	0.25	0	0	0	1	0	0	0.25	0	1	0.25
P3	0.25	0.25	0.25	0	1	0	0	0	1	0.25	0	0	0.25
P4	0.25	0.25	0.25	0	0	0	0	1	0	0.25	0	0	0.25

We spread each annual average across four periods according to the table above, giving us the complete array V_{base} of size 52x64x44.

Each simulation includes a spinup time of random length (8 to 20 periods in this application); these are treated as repeated cycles of the year 2005.

SI2.*1.5.3 Time constraints*
We estimated time constraints specific to layers from each income quartile, for each place, in a two step procedure.

First, we identified quarters in which income sources would demand an agent's time as follows: we assumed income sources 3, 4, 6, and 16 to take time across all four quarterly periods, and consulted an agricultural calendar for Bangladesh (BBS 2017) for approximate sowing and harvest periods, such that quarters in which a layer demands time from an agent, for each layer, is as follows:

Table S8: Quarterly periods requiring effort, by income source

	Rest of other income	Wage income	Salary income	Sugarcane income	Jute income	Wheat income	Boro rice income	Aman rice income	Aus rice income	Oilseed income	Pulse income	Maize income	Income from other crops
P1	1	1	1	1	1	0	1	0	0	1	1	1	1
P2	1	1	1	1	1	0	1	0	1	1	0	1	1
P3	1	1	1	1	0	0	0	1	1	1	0	0	1
P4	1	1	1	1	0	1	0	1	0	1	1	1	1

Second, we calibrated time constraints such that income diversity for agents in each income quartile in the simulation could match that observed in the HIES. Generally speaking, respondents in higher income quartiles in the HIES tended to have more diversified incomes (Figure S9), with the poorest quartile having 0 or 1 sources on average, and the richest quartiles having 3 or 4.

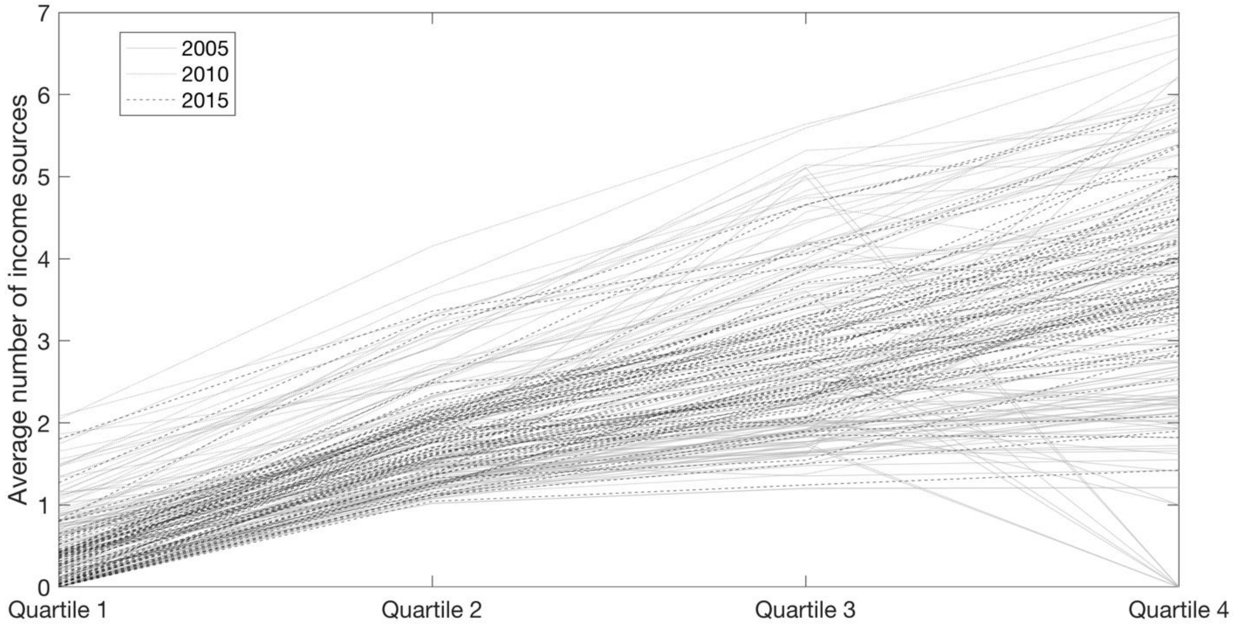

Figure S9: Average number of income sources, by income quartile, in the HIES. Each line represents one district in one wave (2005, 2010, or 2015) of the HIES.

We used the data displayed in Figure S8 to estimate appropriate time constraints for each district, averaging across survey waves to generate one set of average income source counts for each quartile *i*. We specified that for each income source, the *i-1th* income quartile layer was a **prerequisite** (see main ODD for explanation of prerequisites) for the ith layer (e.g., to be a Q3 boro rice earner, an agent must also be occupying Q1 and Q2 for boro rice), and estimated time constraints such that the cumulative time constraint of layers 1 through *i* would allow an agent to occupy the expected number of layers at income quartile *i*, with a small amount of random noise added to the calculation. Consider the following example:

Table S10: Example time constraint calculation along quartiles from same income source

Quartile	Additional time constraint	Cumulative time constraint	Maximum possible layers within time constraint (< 1)
**Q1**	0.95	0.95	1
**Q2**	-0.475	0.475	2
**Q3**	-0.15833	0.31667	3
**Q4**	-0.0792	0.23747	4

In this example, the first-quartile layer for the income source has a time constraint of 0.95 (95% of an agent's time), such that it is the only layer the agent can occupy. The second-quartile layer of the same source has a marginal time constraint of -0.475, such that the combined time constraint is 0.475, allowing up to 2 layers of similar time constraint to be occupied. In the agricultural case, this might represent an agent purchasing more property but also mechanizing and capturing economies of scale (in both time and income, perhaps).

SI2.*1.5.4 Fixed and open layers*
We specified all agricultural layers as 'open,' meaning that there were not a defined number of slots, but that the earned income from each layer may decline (depending on the values *m* and *k* in Eqn. 1) as the number of agents

occupying the layer exceeded those expected (i.e., proportional to the HIES). We specified the layers 'rest of other income' (which we believe to largely be property income), wage income, and salary income as 'fixed,' with a hard number of slots that could not be exceeded:

Table S11: Fixed or open layers, by income source

	Rest of other income	Wage income	Salary income	Sugarcane income	Jute income	Wheat income	Boro rice income	Aman rice income	Aus rice income	Oilseed income	Pulse income	Maize income	Income from other crops
Fixed?	Yes	Yes	Yes	No	No	No	No	No	No	No	No	No	No

For layers that are fixed, agents have a belief about the likelihood of actually gaining a slot, which allows them to estimate the expected utility from that layer. This belief is updated through their social networks (i.e., I heard from my cousin that company X in place Y was hiring; see main ODD for mechanism); agents may choose to migrate based on an expected utility, but in the end not gain access to the layer.

SI2.*1.5.5 Access Costs*

Access costs are coded in MIDAS as a list of discrete cost items that may be associated with one or more layers; in turn, layers may be associated with one or more access costs. This allows representation, for instance, of the cost of multiple certifications in order to be able to practice law in a particular place (and the additional costs at the margin to transfer those credentials to a new place). It also allows representation of immobile assets such as properties, by specifying unique and separate access costs to access a 'property' layer in different places, for example.

In the current application, we include only income streams, but make the following assumption – that the costs of accessing sources 4 and 6 (wage and salary income) allow agents to access those sources in all parts of Bangladesh; i.e., they are transferrable qualifications. All other sources represent earnings from assets (properties in the case of source 3; farm land in all other cases), which we treat as immovable. **The present application does not include the possibility of selling assets to facilitate a move, though this is a possible area for extension if model findings indicate that it is a relevant constraint.**

Rather than try to identify from an additional data source the likely costs of purchasing land, buildings, or accessing wage and salary levels – which could be unavailable or inconsistent – we applied a simple model to generate plausible access costs for each layer, based on an underlying assumption of a reasonable expected rate of return on investment. The fraction of an investment that must be recovered each year of a project in order to break even, known as the uniform series capital cost recovery factor, is defined as:

$$\frac{A}{P} = \frac{i \cdot (1+i)^n}{(1+i)^n - 1} \qquad (2)$$

Where *A* is the annual return in each of *n* project years, *P* is the upfront cost, and *i* is the discount rate. We leverage this model to estimate the reasonable investment costs to earn a return on investment *r* as:

$$P = A \cdot (1+r) \cdot \frac{(1+i)^n - 1}{i \cdot (1+i)^n} \qquad (3)$$

The value of *P* is used as the cost of accessing a layer with average annual return *A*. Values for *r*, *i*, and *n* are estimated as calibration parameters, with the same values applied to all layers in the simulation.

SI2.***1.6 Moving costs***

Moving costs were estimated by calibration, as specified in Section 1.8.

SI2.***1.7 Remittance costs***

Remittance costs were estimated by calibration, as specified in Section 1.8.

SI2.***1.8 Calibration procedure***

We applied an Approximate Bayesian Calibration (ABC) approach over 65 model parameters of varying scope (Table S12), calibrating against the relative inter-district migration rate Q, with flows weighted by the populations of both source and destination districts, as:

$$SSE_Q = \frac{\sum_i \sum_j w_i w_j \left(Q_{ij,modeled} - Q_{ij,observed}\right)^2}{\sum_i \sum_j w_i w_j}$$

where i and j are the source and destination districts, and w is population. We conducted 4 rounds of Monte Carlo simulation across the 65 parameters, with parameter values drawn from a uniform distribution between some minimum and maximum value. At the end of each round, we selected the highest 1% of calibration results, and used the minimum and maximum values of each calibration parameter observed in this set to define the bounds of the uniform distribution for the following round. In total, we conducted 5,373 simulations in Round 1; 2,969 simulations in Round 2; 3,547 simulations in Round 3; and 3,565 simulations in Round 4 (variation across rounds due to priority and speed in the high-performance cluster). The final 1% of calibrations have weighted Pearson Correlations (for relative migration rates) ranging from 0.1752 to 0.2561.

By comparison, in a separate exercise in which income values for the same set of utility layers were replaced with random numbers (i.e., a null model), the same weighted Pearson Correlation had a maximum of 0.0326.

Table S12 - Calibration Parameters

Scope	Parameter	Short Description
model	spinupTime	Time steps run before ‘time 0’ in simulation, for agents to develop expectations about utility layers
	numAgents	Number of agents in simulation
	utility_k	Utility model parameter from Section 1.5.2
	utility_m	Utility model parameter from Section 1.5.2
	utility_noise	Random noise in utility model from Section 1.5.2
	utility_iReturn	Access cost model parameter from Section 1.5.5
	utility_iDiscount	Access cost model parameter from Section 1.5.5
	utility_iYears	Access cost model parameter from Section 1.5.5

	creditMultiplier	Scalar multiple of paid access costs that gives proxy for access to credit, in Submodel 5
	remitRate	Fraction of remitted resources taken as fee
map	movingCostPerMile	Costs per mile to move between two places
	minDistForCost	Shortest distance at which costs accrue
	maxDistForCost	Greatest distance to which costs rise
network	connectionsMean	Mean and standard deviation in number of network connections per agent
	connectionsSD	
	weightLocation	Weight of sharing same location in likelihood of new link forming
	weightNetworkLink	Weight of having shared network connections in likelihood of new link forming
	weightSameLayer	Weight of occupying the same utility layer in likelihood of new link forming
	decayPerStep	Amount by which a network link strength decays in each timestep
	interactBump	Amount by which a network link strength increases due to social interaction
	shareBump	Amount by which a network link strength increases due to sharing of resources
	incomeShareFractionMean	Mean and standard deviation in fraction of new income shared across an agent's social network each time step
	incomeShareFractionSD	
agent	shareCostThresholdMean	Mean and standard deviation in the fraction of overall remittance that is lost to fees, above which agents choose not to share
	shareCostThresholdSD	
	interactMean	Mean and standard deviation in likelihood that agent participates in interactions in a time step
	interactSD	
	meetNewMean	Mean and standard deviation in probability that agent will meet a new agent during a time step
	meetNewSD	
	probAddFitElementMean	Mean and standard deviation in likelihood that algorithm to generate new utility layer portfolios for consideration will try to add an additional layer to a portfolio
	probAddFitElementSD	
	randomLearnMean	Mean and standard deviation in likelihood that agent will randomly
	randomLearnSD	

		learn new information about utility layers outside of a social interaction during a time step
	randomLearnCountMean	Mean and standard deviation in the number of pieces of information that an agent will randomly learn, if the agent learns outside of a social interaction
	randomLearnCountSD	
	chooseMean	Mean and standard deviation in the likelihood that an agent will reconsider their portfolio during a time step
	chooseSD	
	knowledgeShareFracMean	Mean and standard deviation in the fraction of knowledge about utility layers that an agent will share during an interaction
	knowledgeShareFracSD	
	bestLocationMean	Mean and standard deviation in the number of high-utility locations an agent will keep in memory after considering them
	bestLocationSD	
	bestPortfolioMean	Mean and standard deviation in the number of high-utility portfolios an agent will keep in memory for each location it considers
	bestPortfolioSD	
	randomLocationMean	Mean and standard deviation in the number of locations an agent will randomly select for consideration when evaluating new portfolios
	randomLocationSD	
	randomPortfolioMean	Mean and standard deviation in the number of portfolios an agent will generate randomly in each location when evaluating new portfolios
	randomPortfolioSD	
	numPeriodsEvaluateMean	Mean and standard deviation in the number of time periods an agent uses to consider future utility stream when comparing portfolios
	numPeriodsEvaluateSD	
	numPeriodsMemoryMean	Mean and standard deviation in the number of past time periods agent keeps in memory
	numPeriodsMemorySD	
	discountRateMean	Mean and standard deviation in agent-specific discount rate
	discountRateSD	
	rValueMean	Mean and standard deviation in agent-specific constant relative risk aversion coefficient
	rValueSD	
	bListMean	Mean and standard deviation in agent-specific coefficients on utility in different forms (income, etc.)
	bListSD	
	prospectLossMean	Mean and standard deviation in agent-specific disutility of loss relative to utility of gain
	prospectLossSD	

	informedExpectedProbJoinLayerMean	Mean and standard deviation in agent's expectation to be able to join a layer, given that they have information about job openings
	informedExpectedProbJoinLayerSD	
	uninformedMaxExpectedProbJoinLayerMean	Mean and standard deviation in agent's expectation to be able to join a layer, given that they do not have information about job openings
	uninformedMaxExpectedProbJoinLayerSD	
	expectationDecayMean	Mean and standard deviation in the rate at which expectation about job openings decays per time step
	expectationDecaySD	

SI2.***1.9 Analysis of calibration and limitations***

Limitations in our available data (Table S1) necessitate some compromises in our efforts to calibrate the model. We calibrate against the relative inter-district migration rate, averaged over a period of 10 years. We have little data on the economic conditions prior to our first year of migration data, and so have no real basis for attempting to calibrate to any observed trend along these 10 years, and instead choose to average them. We elected not to attempt a cross validation exercise for the model, given that migration rates are highly autocorrelated from year to year and are not independent within a year, such that there is no way to meaningfully split the data without producing a test dataset that is strongly dependent on the training dataset. We have no reason to believe our key, qualitative findings would shift had we pursued a cross-validation exercise rather than a pure calibration exercise.

Our calibration space of 65 parameters is large, but it is worth emphasizing that many of the parameters in Table S12 are elements of the agent decision model that have no real-world parallel, may take only a small number of states in the model, and are not likely to strongly influence results. We include them in the calibration exercise to avoid the small risk of their leading to modeling artifacts in our results, but find overall that only a small number of parameters have a strong impact on our prediction of relative migration rates (Figure S13).

Specifically, five parameters stand out as having larger influence on our outcome of interest. First, the 'credit multiplier,' which scales the amount of credit available to agents, described in detail in Submodel 5. Second, the number of new locations selected randomly for consideration in the algorithm that evaluates alternative livelihood portfolios. Third, the average risk tolerance of an agent. Fourth, the fraction of an agent's knowledge about utility layer histories that is shared with another agent during a social interaction. Fifth, the average likelihood that an agent will choose to consider alternate livelihoods portfolios during a given timestep. We show the distribution of migration outcomes as functions of these 5 parameters across all calibration runs, as well as within the top 1% of runs used in our model projections, in Figure S14. We note that for all 5 of these parameters, strongly performing calibrations occurred along much of the overall ranges selected for the calibration exercise. On the one hand, this alleviates concern of bias introduced by poor selection of ranges – as would be suggested had the strongest calibrations been strongly clustered at either end of the parameter range. On the other hand, it also highlights the equifinality challenge of calibration-driven modeling exercises. While there are patterns observable from Figure S14 (strong calibrations typically offered lower credit, had agents consider more locations, had more risk-tolerant agents, had agents share less information at a time with peers, and had agents re-evaluate their livelihoods more often), there is also much variation across strong calibrations. This indicates that different sets of parameters were able to similarly reproduce our outcome of interest (the equifinality problem). As a simple illustration, consider a sample output from a k-means clustering algorithm tasked with finding two clusters from the distribution of these top 5 parameters (Figure S15). The clusters are not strongly separated (Silhouette values in Figure S15A are not all close to 1) but the 2nd cluster (blue squares in

Figure S15B) offers a narrative that stands out from those calibrations in the first cluster – of agents more likely to re-evaluate their livelihoods and consider more locations, likely to share more information with their peers, but to be less risk tolerant overall and experience lower access to credit overall. We do not make efforts in our analysis to consider these equifinal parameterizations separately, but rather acknowledge that they contribute to an ensemble of plausible agent and simulation parameterizations.

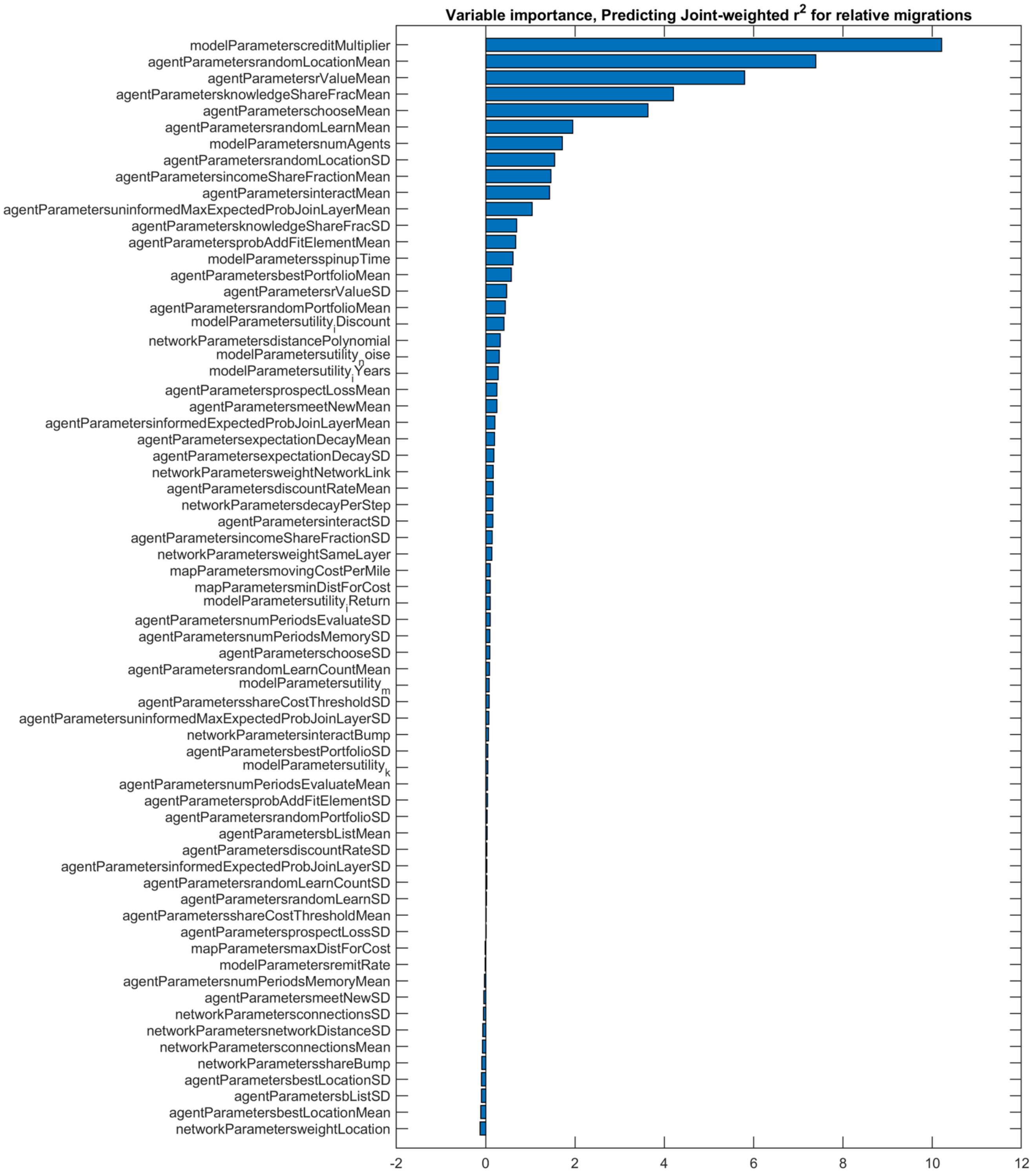

Figure S13: Variable importance measures in predicting relative migration rates across calibration runs

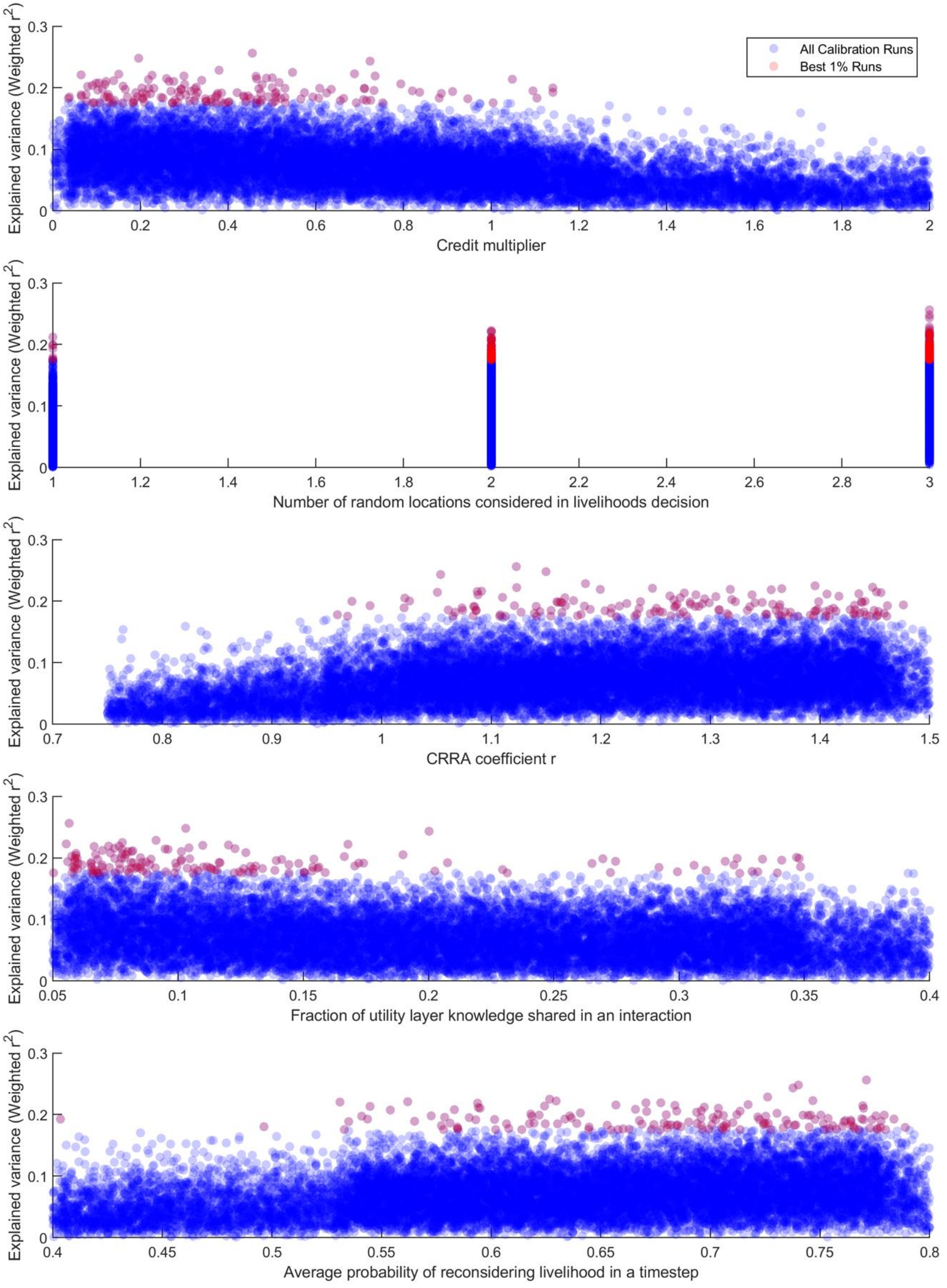

Figure S14: Top 5 important parameters in calibration exercise, and distribution of their values across calibration exercise and in final top 1% of calibrations

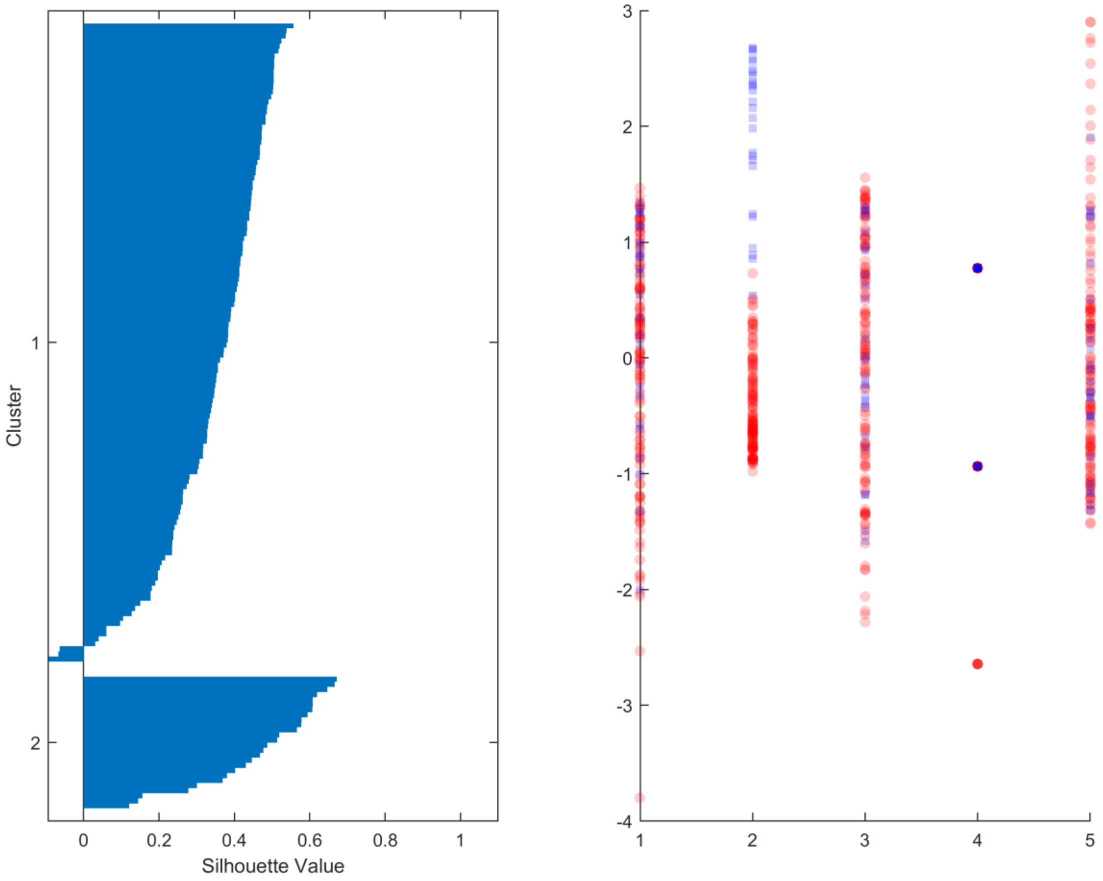

Figure S15: A) Silhouette plot and B) distribution of top 5 parameter values (z-scores) for sample k-means cluster output using 2 clusters. Values in panel B X-axis indicate 1) the average probability of reconsidering livelihoods within a timestep; 2) the fraction of utility layer knowledge shared in an interaction; 3) the average risk tolerance of an agent; 4) the number of locations randomly selected for consideration in livelihoods decision; and 5) the credit multiplier.

SI2.**2 Projections of inter-district migration to 2100 under anticipated sea-level rise**

We conducted a set of experiments using our calibrated model application to estimate the effects of flooding (via impacts to income) on inter-district migration out to 2100 under three of the representative concentration pathways (RCPs; 2.6, 4.5, and 8.5).

Sea level rise is not globally uniform, with considerable gradients driven by non-climatic local factors such as glacial isostatic adjustment and tectonics. Here we employ local relative SLR projections from (Kopp et al. 2017), which account for these, among other time- and geographically-varying, components. These projections are conditional on Representative Concentration Pathways (RCPs) 2.6, 4.5, and 8.5 (IIASA, 2009), and treat 2000 as the baseline year where SLR=0.

To assess extreme flood event probabilities, we use local return level curves from the Global Tides and Surge Reanalysis (Muis et al. 2016). These curves define the local annual probability P(X > h) of at least one flood

exceeding height h in the year 2000 relative to local mean sea level (MSL). Given the assumption that return level curves will not change this century, given a SLR projection SLR(y) for year y, we can estimate the future annual probability of a flood exceeding h as P(X> (h-SLR(y)). Using mean higher-high water (MHHW) deviations from MSL provided by Mark Merrifield, University of Hawaii, developed using the model TPX08 (Egbert and Erofeeva 2002), we compute this function across the entirety of Bangladesh at water heights 0-5m at 0.1m intervals above MHHW, for decades between 2000 and 2100, and under RCPs 2.6, 4.5, and 8.5.

We used the above function to instantiate plausible annual peak floods for Bangladesh. For each year in a simulation we took one random uniform draw between 0 and 1, and used this number to estimate the peak flood depth in each district. Draws were independent between years, so that inter-district flooding is highly correlated within a year but any autocorrelation over time occurs only due to correlation in likelihoods.

We predicted the future economy for Bangladesh out to 2100 by randomly drawing one of our three periods of available income data (2005, 2010, and 2015) with replacement at five-year intervals from 2020 to 2100, interpolating for the years in-between. We used a damage function for flood depth on agricultural and non-agricultural income derived from Quisumbing and Mueller (2011), as follows:

	% drop in income per foot of flood shock, same year	% drop in income per foot of flood shock, following year
Agricultural income	45	2.5
Non-agricultural income	0	13.8

Because this damage function is defined based on the relative concept of ‘flood shock,’ and not the absolute concept of flood depth, we included a parameter for the ‘normal flood depth’ (as a fraction of the statistical expected flood depth) in order to calculate a flood shock (as total flood minus normal flood depth). The value of this parameter is unknown, so that our simulation experiments included a sensitivity analysis on this parameter – a random value between 0 and 1.5 (e.g., up to 150% of statistical expected flood depth) was drawn for every simulation run.

We conducted a total of 871 simulation runs across RCP 2.6 (255 runs), RCP 4.5 (294 runs), and RCP 8.5 (322 runs). Relative importance of parameters in shaping migration outcomes was estimated using Matlab’s treebagger algorithm using forests of 1000 randomly generated regression trees; parameter importance is estimated by the relative increase in prediction error when that parameter is excluded from the generation of trees, compared to the error rate when it is included in the generation of trees.

SI2.3 Literature Cited

BBS. 2017. *Yearbook of Agricultural Statistics-2016*. Dhaka, Bangladesh.

Bert, F. E., G. P. Podestá, S. L. Rovere, Á. N. Menéndez, M. North, E. Tatara, C. E. Laciana, E. Weber, and F. R. Toranzo. 2011. An agent based model to simulate structural and land use changes in agricultural systems of the argentine pampas. *Ecological Modelling* 222(19):3486–3499.

Dave, C., C. Eckel, C. Johnson, and C. Rojas. 2010. Eliciting Risk Preferences: When is Simple Better ? *Journal of Risk and Uncertainty* 41(3):219–243.

Egbert, G. D., and S. Y. Erofeeva. 2002. Efficient inverse modeling of barotropic ocean tides. *Journal of Atmospheric and Oceanic Technology* 19(2):183–204.

Feder, G. 1980. Farm Size , Risk Aversion and the Adoption of New Technology under Uncertainty. *Oxford Economics Papers* 32(2):263–283.

Hong, G. 2015. Examining the role of amenities in migration decisions: A structural estimation approach. *Papers in Regional Science* 95(4).

Kahneman, D. 2003. Maps of Bounded Rationality: Psychology for Behavioral Economics. *The American Economic Review* 93(5):1449–1475.

Kopp, R. E., R. M. DeConto, D. A. Bader, C. C. Hay, R. M. Horton, S. Kulp, M. Oppenheimer, D. Pollard, and B. H. Strauss. 2017. Evolving Understanding of Antarctic Ice-Sheet Physics and Ambiguity in Probabilistic Sea-Level Projections. *Earth's Future* 5(12):1217–1233.

Minnesota Population Center. 2015. Integrated Public Use Microdata Series, International: Version 6.4 [dataset]. University of Minnesota, Minneapolis, MN.

Moon, B. 1995. Paradigms in migration research: exploring "moorings" as a schema. *Progress in human geography* 19(4):504–524.

Muis, S., M. Verlaan, H. C. Winsemius, J. C. J. H. Aerts, and P. J. Ward. 2016. A global reanalysis of storm surges and extreme sea levels. *Nature Communications* 7(May).

Müller, B., F. Bohn, G. Dreßler, J. Groeneveld, C. Klassert, R. Martin, M. Schlüter, J. Schulze, H. Weise, and N. Schwarz. 2013. Describing human decisions in agent-based models - ODD+D, an extension of the ODD protocol. *Environmental Modelling and Software* 48:37–48.

Quisumbing, A., and V. Mueller. 2011. How Resilient are Labour Markets to Natural Disasters? The Case of the 1998 Bangladesh Flood. *Journal of Development Studies* 47(12):1954–1971.

Rubenstein, A. 1998. *Modeling Bounded Rationality*. Page (K. G. Persson, editor) *Southern Economic Journal*. MIT University Press, Cambridge, MA.

Schlüter, M., A. Baeza, G. Dressler, K. Frank, J. Groeneveld, W. Jager, M. A. Janssen, R. R. J. Mcallister, B. Müller, K. Orach, N. Schwarz, and N. Wijermans. 2017. A framework for mapping and comparing behavioural theories in models of social-ecological systems. *Ecological Economics* 131:21–35.

Stimson, R. J., and R. McCrea. 2004. A push-pull framework for modelling the relocation of retirees to a retirement village: The Australian experience. *Environment and Planning A* 36(8):1451–1470.

UNDESA. 2013. World Fertility Data 2012.

WHO. 2015. Global Health Observatory data repository.

PART SI3 - ODD+D Description – The Migration, Intensification, and Diversification as Adaptive Strategies (MIDAS) model framework

This document follows the ODD+D protocol to describe agent-based models with individual decision-making (Müller et al. 2013). This document describes properties of the MIDAS framework, while refraining from describing specific implementations (models) constructed from the MIDAS framework.

SI3.I OVERVIEW

SI3.I.i Purpose

SI3.*I.i.a What is the purpose of the study?*

The purpose of this model (hereon, MIDAS) is to simulate livelihoods decision making at the individual and household level, including intensification (focus on a small number of activities for income), diversification (spreading across a larger number of activities for income), and migration (changing locations to access opportunities for income), as functions of available opportunities varying in space and time.

Examples of appropriate questions for an application of MIDAS might include:

- How important are household social ties in mitigating income shocks?
- What kinds of social programs can best help families adapt to long-term agricultural droughts?
- How can the spread of urban services to rural areas stem the flow of rural-to-urban migration?
- How does geographic dispersion benefit communities?

SI3.*I.i.b For whom is the model designed?*

This model is designed for researchers in the area of livelihoods, migration, and household decision-making or risk management.

SI3.I.ii Entities, state variables and scales

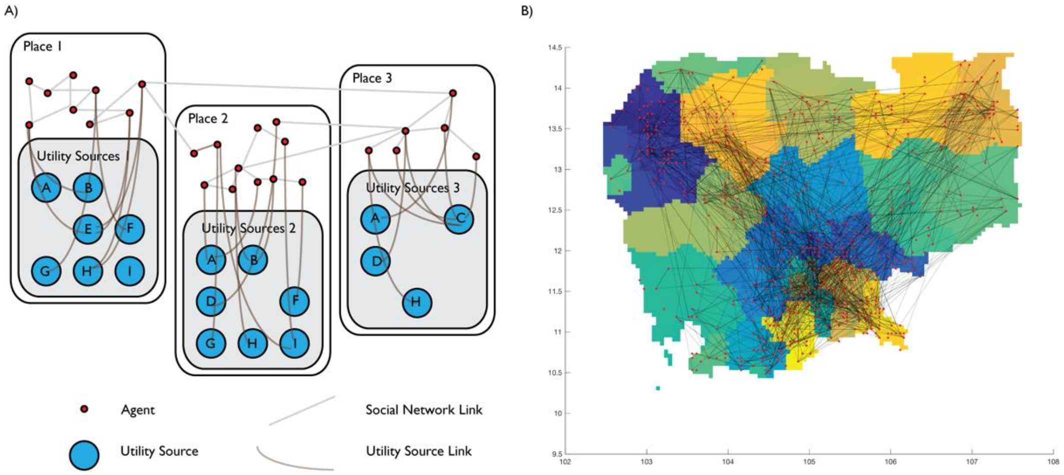

Figure S16: A) Agents located in places, embedded in a social network, and drawing from utility sources as in the MIDAS framework; B) Sample visualization of agents in a two-dimensional representation of Cambodia in a MIDAS simulation.

SI3.*I.ii.a What kinds of entities are in the model?*

MIDAS models individual agents deriving livelihoods, connected to each other by social networks, and existing at particular 'places' – points in a two-dimensional space.

SI3.*I.ii.b By what attributes (i.e. state variables and parameters) are these entities characterized?*

Agents are described by the parameters outlined in Table S17.

The social network links between any two agents *i* and *j* are described by two network strength parameters S_{ij} and S_{ji}, denoting the closeness of each one-way relationship.

Places (or nodes) are described by a location in two-dimensional space, a set of administrative identifiers of variable length (e.g., country, state, county, district, etc.), the potential value of any utility layers included in the simulation at that place, as well as the set of particular costs an agent must assume in order to access that utility layer at that place (Figure S16A).

The values taken by utility layers may be provided as input to the model (i.e., fully exogenous), determined internally by model properties (i.e., fully endogenous) or some mix of both.

SI3.*I.ii.c What are the exogenous factors/drivers of the model?*

The universe of available utility layers (which may represent income opportunities, assets, or any other concept from which agents may derive utility) is exogenous to the model. The values taken in space and time for each of these layers may be partially or fully exogenous to the model. Additionally, the location of places occupied by the agents, and the structure of the social network among agents, are determined exogenously at initialization.

Table S17– Agent Parameter List

	Parameter	Description
Determined on initialization	**id**	Identifier for each agent
	**Gender**	Gender of agent
	**incomeShareFraction**	Fraction of income to the agent shared across social network in a given timestep
	**shareCostThreshold**	Fraction of the overall amount of a remittance lost to transaction costs, above which the agent will choose not to make that share
	**knowledgeShareFrac**	Fraction of their accumulated knowledge (of opportunities in other places) shared with agents during social interaction
	**pInteract**	Likelihood of current agent to interact with other agents to exchange information in a given timestep
	**pMeet**	Likelihood of current agent to meet a new agent in a given timestep
	**pChoose**	Likelihood of current agent to make a decision about income portfolio in a given timestep
	**pRandomLearn**	Likelihood of current agent to learn new information about income opportunities randomly in a given timestep
	**countRandomLearn**	Number of new pieces of information learned randomly, if agent learns randomly during a timestep
	**numBestLocation**	Number of good node/locations agent will retain in memory from previous decision making
	**numBestPortfolio**	Number of good portfolios from a given location agent will retain in memory from previous decision making
	**numRandomLocation**	Number of node/locations agent will draw randomly in decision making
	**numRandomPortfolio**	Number of portfolios in a given location agent will draw randomly in decision making
	**numPeriodsEvaluate**	Number of time periods over which agent will evaluate and compare different portfolios when making a decision
	**numPeriodsMemory**	Number of time periods of past experience agent will hold in memory to inform decisions
	**discountRate**	Agent's individual discount rate on future time periods
	**rValue**	Agent's individual constant relative risk aversion coefficient
	**bList**	Agent’s individual utility preferences on different forms of utility (income, use value, etc.) available in the simulation
	**pA**	Agent’s highest expectation of accessing a layer given that a slot is known to be available
	**pB**	Agent’s highest expectation of accessing a layer when it is not known whether a slot is avaiable
Updated endogenousl	**location**	Identifier for the specific node/location where the agent is located
	**age**	Age of agent (determined upon initialization and then updated)
	**wealth**	Cumulated wealth of the agent
	**incomeLayersHistory**	Agent's cumulated knowledge of past income opportunities (through experience, random learning, and social interaction)

**bestPortfolios**	Retained list of income portfolios in various locations considered during previous decisions
**accessCodesPaid**	List of costs already accrued by the agent to access particular layers (e.g., teaching licenses, necessary equipment, etc.)
**currentPortfolio**	Agent's current portfolio of income layers accessed
**network**	List of other agents to which current agent has a social connection, with link strength from 0 to 1 (determined upon initialization and then updated)
**pChild**	Likelihood of current agent to have a child in a given timestep, informed by age-specific fertility data
**pDie**	Likelihood of current agent to die in a given timestep, informed by age-specific mortality data
**pOpening**	Agent expectation of layers in different places having available openings

SI3.*I.ii.d If applicable, how is space included in the model?*

Agents occupy specific 'places' or nodes in two-dimensional space, with each node representing a hub of utility opportunities that agents may access, and in some cases (such as where the values of utility layers are defined by the user as density dependent) compete for. Depending on the definitions in input data, these points may represent villages, cities, counties, states, or other administrative divisions.

SI3.*I.ii.e What are the temporal and spatial resolutions and extents of the model?*

MIDAS is built with a flexible timescale and temporal resolution. There is an underlying 'timestep' within which MIDAS will cycle once through all agents and allow them to act, and may also evaluate and update utility. This underlying timestep could be daily or weekly (as in wages), seasonally (as in harvests), or any other division the modeler deems appropriate. There are several other implicit timescales that are loosely coupled to this base timestep during a model run: i) social interactions, ii) portfolio evaluations, and iii) random learning. Each agent has an individual-specific likelihood of participating in each of these processes, tested during each timestep. In this manner, the exchange of information or the making of life choices is not spuriously coupled to the regular passage of time – some agents may interact regularly while others do not, some may make changes to their income portfolios often, others seldom. The evaluation of utility occurs at regular multiples of the underlying timestep. With this structure, MIDAS can create conditions for work-week commuting, seasonal migration, as well as long-term patterns of immigration.

The spatial resolution and extent of the model is defined exogenously by the scope of input map data.

SI3.I.iii Process overview and scheduling

SI3.*I.iii.a What entity does what, and in what order?*

In a single timestep, MIDAS first loops once through all agents in a newly randomized ordering, testing whether they participate in communication with other agents, random learning, or whether they update decisions about their best utility portfolio (Figure S15). Following this agent loop, if utility is to be evaluated and received by

agents in the current timestep, MIDAS takes an additional loop to do so – calculating utility first, then looping through each agent to receive new utility and share across their social networks (Figure S18).

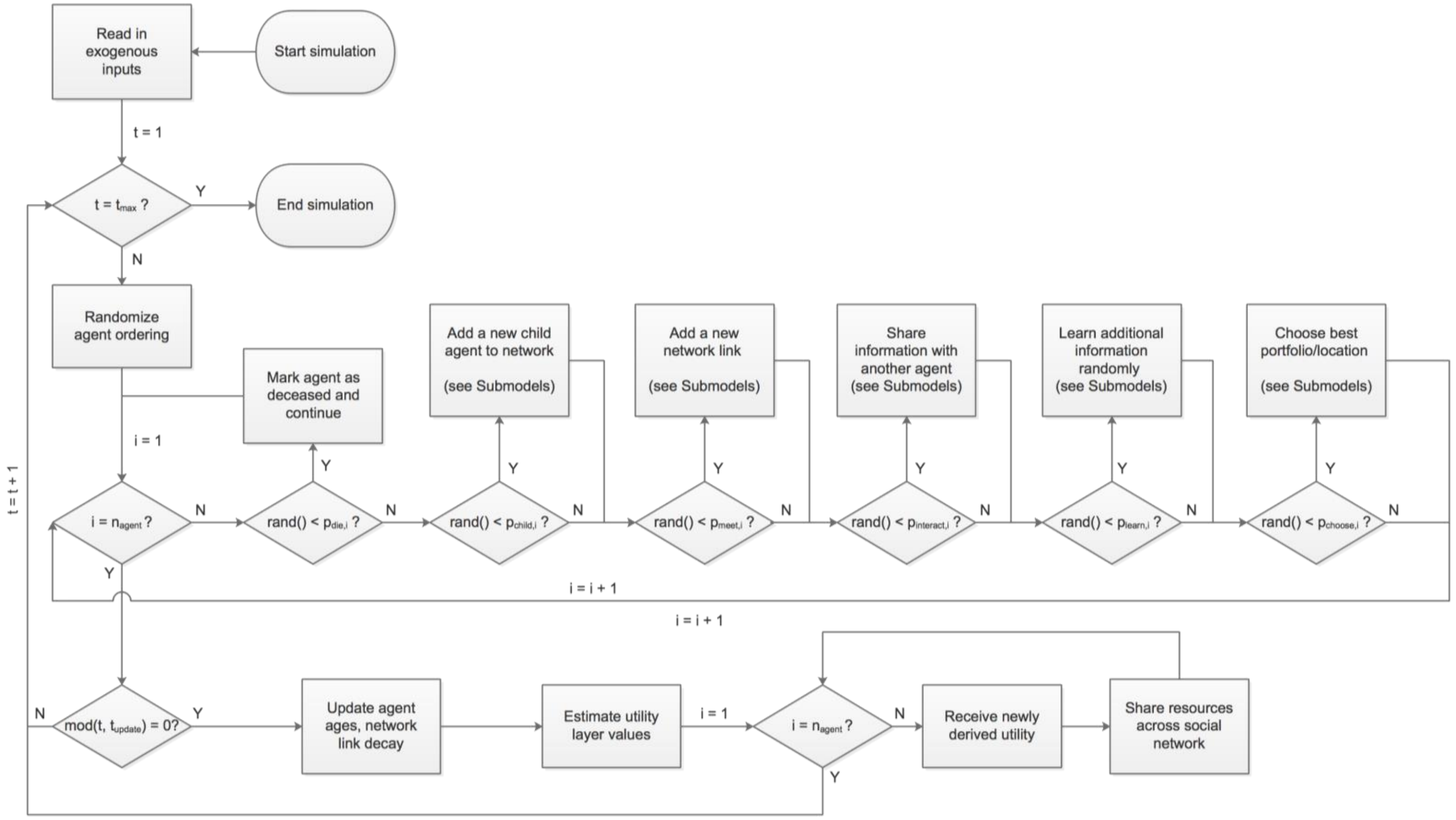

Figure S18: MIDAS process flow overview. Agent information sharing, learning, and decision flow diagrams included in Submodels section.

SI3.II DESIGN CONCEPTS

SI3.II.i Theoretical and Empirical Background

SI3.*II.i.a Which general concepts, theories or hypotheses are underlying the model's design at the system level or at the level(s) of the submodel(s) (apart from the decision model)? What is the link to complexity and the purpose of the model?*

There are no particular literatures that inform the system-level or submodel behavior of MIDAS, outside of the decision model. Many of the modeler's assumptions regarding system-level behavior will be implicit in the utility layers provided as input to a specific implementation of MIDAS, which can represent income, assets, or any other aspect of an agent's environment from which it might derive utility. Spatiotemporal patterns in these input layers, or equations governing their calculation, will embed specific modeling assumptions regarding system behavior and should be described in each instantiation.

SI3.*II.i.b On what assumptions is/are the agents' decision model(s) based?*

The decision model used in the current MIDAS version (described below in II.ii and in Submodels) embeds the following assumptions with roots in decision literature:

- Factors shaping livelihoods decisions (of which migration is one) are well described by 'pushes' (declines in opportunity local to the agent), 'pulls' (availability of opportunities distant from the agent), and 'moorings' (investments and ties – such as assets or family – that cannot be easily moved with the agent) (Moon 1995, Stimson and McCrea 2004).
- Decisions regarding portfolios of livelihoods opportunities are boundedly rational, such that only a small number of possible competing opportunities are considered at one time (Rubenstein 1998, Kahneman 2003).
- Given two income streams of equal nominal value, a risk-averse agent should prefer the less time-varying of the two, such that a livelihoods portfolio that spreads activities across activities with uncorrelated risks (diversification) can be a risk minimization strategy; this can be accomplished via an expected utility framework (Feder 1980, Bert et al. 2011, Hong 2015) based on constant relative risk aversion (Dave et al. 2010).
- In considering future income streams, agents will dislike losses approximately twice as strongly as they enjoy equivalent games, as in prospect theory (Kahneman 2003, Schlüter et al. 2017).

SI3.*II.i.c Why is/are certain decision model(s) chosen?*

The set of assumptions outlined above that bound the decision model have been chosen in order that: i) migration can emerge as an adaptive strategy alongside other livelihoods strategies (such as diversification or intensification) without being hard-coded in (e.g., as a stage-wise decision), ii) preferences for crop diversification along a season, or commuting/local migration over short periods, can emerge in the same modeling space, and iii) factors outside of income, such as assets or preferences for family connection, can shape livelihoods decision-making (conditional on the availability of data on relative preferences).

SI3.*II.i.d If the model/submodel (e.g. the decision model) is based on empirical data, where do the data come from?*

This document describes the MIDAS framework rather than any specific implementation within it. In general, utility layers should be informed either by primary data collection or secondary sources such as censuses or living standards surveys. The relative value to the agent of those different layers (when they do not directly show income, but use value or existence value) should be informed by experimental means (such as discrete choice experiments).

SI3.*II.i.e At which level of aggregation were the data available?*

In general, the aggregation scale of availability of secondary data (like income) will constrain the scale of the model. Secondary income data are commonly available from census or representative sample surveys down to state or county levels.

SI3.II.ii Individual Decision-Making

SI3.*II.ii.a What are the subjects and objects of the decision-making? On which level of aggregation is decision-making modeled? Are multiple levels of decision making included?*

Individual agents make decisions on the best portfolio of utility-yielding activities (including income sources, assets, as well as any other feature from which an agent may derive utility over time) across a sample of different places, including their current place and current portfolio.

SI3.*II.ii.b What is the basic rationality behind agent decision-making in the model? Do agents pursue an explicit objective or have other success criteria?*

Agents pursue a strategy of maximizing expected utility over time.

SI3.*II.ii.c How do agents make their decisions?*

Agents first select a sample of places in the modeled space for which they have some information about utility opportunities, and then select a sample of portfolios in each place. An agent-specific fraction of these places (and portfolios within places) are remembered from previous decision-making; the remaining fraction is drawn randomly. Drawing from their knowledge of past values from these utility layers, agents estimate the future utility stream (converted to a net present value) that would be derived from these portfolios, including any initial costs to move to new locations or access new utility layers, using an expected utility modeling framework (See *Submodels*).

SI3.*II.ii.d Do the agents adapt their behavior to changing endogenous and exogenous state variables? And if yes, how?*

Agents maintain a finite memory of past observation and shared information, so that as utility of different layers changes over time and as they learn more information, their evaluation of expected utility will change.

SI3.*II.ii.e Do social norms or cultural values play a role in the decision-making process?*

Not explicitly in the version of MIDAS presented here.

SI3.*II.ii.f Do spatial aspects play a role in the decision process?*

The initial costs associated with income streams will include moving costs if appropriate, and the costs of sharing resources across the social network are location dependent, thus shaping the expected utility stream of a portfolio.

SI3.*II.ii.g Do temporal aspects play a role in the decision process?*

Timesteps in MIDAS are defined as occurring in 'cycles' that are known to agents (e.g. 12 steps in a 'year' of months or 3 steps in a 'year' of seasons. Agents consider cycles in estimating future utility streams, preserving cycles of estimated value along time and across layers wherever their 'memories' permit (in order to capture any correlation over time across layers).

Agents age over time, and MIDAS allows for agent age to affect a range of agent parameters (such as time horizon for decision making) via scripts specific to an implementation of MIDAS. Agents may die or have children, following age-specific mortality data. Additionally, agent social networks decay over time, and will weaken unless they are enhanced again by interactions or the sharing of remittances

SI3.*II.ii.h To which extent and how is uncertainty included in the agents' decision rules?*

In the present version of MIDAS, utility values are specific to a time and place, but not to an agent – all agents accessing a layer at the same place and time receive the same value, without uncertainty or variation. Future versions may have agent-specific multipliers, but this is not currently an object of inquiry. The benefit of this structure is improved memory management and scalability, as the history of incomes is stored in only one place.

Uncertainty manifests in decision-making only by way of agents' incomplete knowledge of historic/future utility streams, such that their estimations (constructed with existing memory and filling in gaps as best as they can) are imperfect guesses at what the future stream may be.

SI3.II.iii Learning

SI3.*II.iii.a Is individual learning included in the decision process? How do individuals change their decision rules over time as consequence of their experience?*

In the present version of MIDAS, the decision rule remains fixed. Agents build out their best estimate of future utility streams based on their current knowledge, so that as they learn more (by doing, interacting, or learning randomly) their estimations of the value of different portfolios will change. Additionally, as they consider different portfolios and retain their memories of good options, the quality of portfolios compared in subsequent decisions is improved.

SI3.*II.iii.b Is collective learning implemented in the model?*

Agents share elements of their own knowledge with other agents in their social network via social interaction.

SI3.II.iv Individual Sensing

SI3.*II.iv.a What endogenous and exogenous state variables are individuals assumed to sense and consider in their decisions? Is the sensing process erroneous?*

Agents consider their past history of experience and knowledge with the utility layers available in the model, known costs of accessing those layers, known costs of moving, past experience of receiving other support across their social networks and known costs of sharing across their social networks. This process is not erroneous.

SI3.*II.iv.b What state variables of which other individuals can an individual perceive? Is the sensing process erroneous?*

Agents remember sharing across their social networks, without error. However, this is an imperfect prediction of future structure and sharing across their social networks.

SI3.*II.iv.c What is the spatial scale of the sensing?*

Agents are able to learn randomly about past history at any place in the modeled space, and as well can learn any aspects of past knowledge from members of their social network.

SI3.*II.iv.d Are the mechanisms by which agents obtain information modeled explicitly, or are individuals simply assumed to know these variables?*

Agents are assumed to know the costs of accessing utility layers, the costs of moving and the costs of sharing across their social networks. Information regarding utility layer values at particular places and times is learned through experience, sharing of information via social interaction, and random learning.

SI3.*II.iv.e Are the costs for cognition and the costs for gathering information explicitly included in the model?*

There are no explicit search costs in this version of MIDAS, nor is exploration modeled as a decision. It is modeled as a probabilistic process, such that costs, willingness and ability to search are implicit in the likelihood an agent has to interact socially or learn randomly.

SI3.II.v Individual Prediction

SI3.*II.v.a Which data do the agents use to predict future conditions?*

Agents use their knowledge of past utility values, in different places and times, to predict future conditions. To the extent possible, agents preserve the time structure of past knowledge across utility layers, in order to capture patterns across layers and along time.

SI3.*II.v.b What internal models are agents assumed to use to estimate future conditions or consequences of their decisions?*

Agents implement an expected utility model of future utility streams.

SI3.*II.v.c Might agents be erroneous in the prediction process, and how is it implemented?*

Agents are only erroneous to the extent that past performance (and their incomplete knowledge of it) is an imperfect prediction of the future.

SI3.II.vi Interaction

SI3.*II.vi.a Are interactions among agents and entities assumed as direct or indirect?*

Agents interact directly via social interactions that include the exchange of knowledge of past utility, and by sharing their received utility across their social networks.

SI3.*II.vi.b On what do the interactions depend?*

Interactions depend on i) the structure of social networks, including a network link 'strength' that is one way (i.e., agent *i* can have a stronger/weaker connection to agent *j* than agent *j* has to agent *i*); ii) the cost of sharing between the places occupied by any two agents in a social network; iii) and the agent-specific probabilities of participating in a social interaction in a given timestep.

SI3.*II.vi.c If the interactions involve communication, how are such communications represented?*

Communication between agents happens in two places. In social interactions, an agent shares a fraction of their cumulative knowledge of past utility values (drawn randomly) with the other agent in the interaction. In sharing resources, agents have an agent-specific share of their received utility that they share across their social network, proportional to the strength of the network link they share to those agents. Within that interaction, there is an agent-specific threshold fraction of the overall 'remittance' that the agent is willing to spend on the cost of remitting; when this cost is exceeded, the agent simply does not share the resource with that agent.

SI3.*II.vi.d If a coordination network exists, how does it affect the agent behavior? Is the structure of the network imposed or emergent?*

The structure of social networks is imposed at initialization, and is allowed to evolve over the length of the simulation. At both initialization and during the simulation, new links to the current agent are formed by applying weights to all other agents not in the current agent's network, according to factors like i) whether the agent is in the same place as the current agent, ii) whether the agent has common social network connections to the current agent, and iii) whether the agent occupies the same layer (in the same place) as the current agent. Such weights will be implementation-specific, and agents with greater weight will be more likely to be selected to be a new link (see Submodels).

SI3.II.vii Collectives

SI3.*II.vii.a Do the individuals form or belong to aggregations that affect and are affected by the individuals? Are these aggregations imposed by the modeler or do they emerge during the simulation?*

Individual agents are embedded in social networks, among agents that may be in the same or in distant places, and with each directional link described by a 'strength'. At present, these links do not evolve along the length of the simulation, but they may be allowed to do so in future versions. 'Households' may be created through parameterization of social networks, constructed as small cliques of agents with close, strong network links. Future versions of MIDAS in which agents may join the model (or be born), or in which network links may otherwise change (through working together, sharing resources or information), will be able to simulate emergent households.

SI3.*II.vii.b How are collectives represented?*

Collectives are represented only as a set of social network linkages.

SI3.II.viii Heterogeneity

SI3.*II.viii.a Are the agents heterogeneous? If yes, which state variables and/or processes differ between the agents?*

At initialization, agents differ in a number of the key parameters listed in Table 1: incomeShareFraction, shareCostThreshold, knowledgeShareFrac, pMeet, pChild, pDie, pInterac, pChoose, pRandomLearn, countRandomLearn, numBestLocation, numBestPortfolio, numRandomLocation, numRandomPortfolio, numPeriodsEvaluate, numPeriodsMemory, discountRate, bList, and rValue. These values are all drawn from normal distributions and rounded to integers as appropriate. Additionally, agents will differ in the number of social network connections and the strength of their linkages.

Agents will also accumulate knowledge and make decisions differently along the course of the simulation.

SI3.*II.viii.b Are the agents heterogeneous in their decision-making? If yes, which decision models or decision objects differ between the agents?*

Agents do not differ in the structure of their decision-making, only in the decision-related parameters outlined above.

SI3.II.ix Stochasticity

SI3.*II.ix.a What processes (including initialization) are modeled by assuming they are random or partly random?*

In initialization, agent properties and locations, initial utility layer uses, as well as social network links and strengths are drawn stochastically. Along the simulation, agent ordering is randomized in each timestep, and agent participation in social interaction, random learning, or decision-making is also stochastic.

SI3.II.x Observation

SI3.*II.x.a What data are collected from the ABM for testing, understanding and analyzing it, and how and when are they collected?*

This document describes the MIDAS framework, and specific implementations may opt to extract different outcomes. Examples of useful outcomes from MIDAS simulations include the distribution of agents' overall wealth, the rates of in- and out-migration at all administrative levels, the degree of diversification in income and overall livelihoods of individual agents, and degree of diversification of economies of places.

SI3.*II.x.b What key results, outputs or characteristics of the model are emerging from the individuals? (Emergence)*

Migration rates, place-specific economic productivity, and livelihoods diversity of individuals, 'households,' and communities are examples of system-level outcomes emerging from individual decisions.

SI3.III DETAILS

SI3.III.i Implementation Details

SI3.*III.i.a How has the model been implemented?*

The current version of MIDAS is implemented in Matlab, compatible at least up to version 2017a.

SI3.*III.i.b Is the model accessible, and if so where?*

Model code and documentation is available at Github, via DOI: 10.5281/zenodo.154738

SI3.III.ii Initialisation

SI3.*III.ii.a What is the initial state of the model world, i.e. at time t = 0 of a simulation run?*

At initialization, the present version of MIDAS assigns new agents to places proportionally based on population estimates (or any table that assigns relative weights to each place). Agents are randomly assigned to access utility layers available in their current place, and have no memory of previous utility values. Agents will occupy these layers and places, having social interactions and learning randomly, without examining other potential portfolios for a period of t_{spinup}, accumulating information about other layers and places.

SI3.*III.ii.b Is the initialization always the same, or is it allowed to vary among simulations?*

Matlab's random number generator can be seeded to reproduce identical results; otherwise, each new simulation run will be different, as agent generation (and placement) is stochastic.

SI3.*III.ii.c Are the initial values chosen arbitrarily or based on data?*

Population data and utility layers can be drawn from available data or assigned arbitrarily; distributions for agent parameters as well may be informed by experimental data or defined by calibration.

SI3.III.iii Input Data

SI3.*III.iii.a Does the model use input from external sources such as data files or other models to represent processes that change over time?*

Utility layers may be exogenous in full or in part, and may represent scenarios of time variant processes. Additionally, data on agent fertility and mortality, the costs of moving and sending remittances, and any other data that better describe the model context may be added via scripts.

SI3.III.iv Submodels

SI3.*III.iv.a What, in detail, are the submodels that represent the processes listed in 'Process overview and scheduling'?*

Submodel 1 – Childbirth. This decision process is best described by the pseudocode below:

- Female agent *i* gives birth to a new agent *k* that is randomly male or female
- Agent *k* is added to the network with (initially) only the parent as the social network (but this network can evolve over the simulation)

Submodel 2 – New network connection. This decision process is best described by the pseudocode below:

- All agents $j \neq i$ not in the social network of agent *i* are weighted according to factors shaping the likelihood of meeting, by:

$$W_{j \neq i} = \sum_{m} w_m \cdot S_m$$

  where w_m is the weight assigned to factor m (which might be distance, number of shared social connections, etc.) and S_m is the implementation-specific score assigned to factor m (factors might all be rescaled to vary from 0 to 1, for example).
- MIDAS creates a cumulative summation of these weights, and normalizes by the sum of all weights, creating a vector spanning 0 to 1
- A new link is selected by drawing a random number between 0 and 1, and selecting the agent corresponding to the first value in the normalized cumulative sum vector that is larger than that random value

Submodel 3 – Social interaction. This decision process is best described by the pseudocode below:

- Agent *i* chooses a random member agent *j* of social network

- Identify non-overlapping knowledge sets of past utility layers (across places and times) for agents *i* and *j*
- Agent *i* shares $f_{share\ fraction,\ i}$ of knowledge held by agent *i* but not agent *j*, with agent *j*
- Agent *j* shares $f_{share\ fraction,\ j}$ of knowledge held by agent *j* but not agent *i*, with agent *i*

Submodel 4 – Random learning. This decision process is best described by the pseudocode below:

- Agent *i* learns $n_{randomLearn,\ i}$ pieces of information (e.g., specific utility values from a particular layer, place, and time), drawn randomly.

Submodel 5 – Livelihoods decision. This decision process is best described by the pseudocode below:

- Choose a set of locations to consider – $n_{best,loc}$ locations with good options from previous decision-making, and $n_{random,loc}$ locations chosen randomly, as well as the current location.
- For each location,
  - Choose a set of n_{total} portfolios - $n_{best,port}$ portfolios with high value remembered from previous decision-making (if available), and max(n_{total} - $n_{best,port}$, $n_{random,port}$) locations chosen randomly, plus the current portfolio in the case of the current location
    - If any elements of the randomly selected portfolios have 'prerequisites' – layers that must also be accessed in order to access the selected layer
  - For each portfolio,
    - Attempt to estimate a future income stream of utility from the elements of the portfolio, over the upcoming $n_{evaluate}$ periods, preserving structure in time and across layers where possible. Start by drawing a random starting point in agent's memory from the same point in the cycle (e.g., draw from 'Januarys' in memory in order to estimate for an upcoming 'January'), filling in all points in the estimated stream that can be filled by stepping forward from this point in memory, then randomly selecting remaining gaps one by one, filling in in the same way, until there is no further potential to fill in gaps in the estimated stream.
    - Add in expected receipts from across social network, assuming current agent locations and expected costs of sharing
    - Add in any expected costs to access this layer to the first period of the stream, if they have not already been paid (e.g., a car might be a required cost across several layers, while a teaching license would apply specifically to a teaching income layer)
    - Add expected moving costs to the first period of the stream, if the agent is not presently in this location
    - Convert to expected utility, and discount future streams to the present, using the model:

$$U_{k,m} = \begin{cases} \sum_t \left[\dfrac{\left(\beta_{1,k}B + \beta_{2,k}D\right)^{1-r_k}}{1-r_k}\right]^{-(1+d_k)^t} & ;\beta_{1,k}B + \beta_{2,k}D \geq 0 \\ P \cdot \sum_t \left[\dfrac{\left[\text{abs}(\beta_1 B + \beta_2 D)\right]^{1-r_k}}{1-r_k}\right]^{-(1+d_k)^t} & ;\beta_{1,k}B + \beta_{2,k}D < 0 \end{cases}$$

where U is the overall present 'utility value' of the stream, *r* is a coefficient capturing agent *k*'s constant relative risk aversion, and *d* is agent *k*'s discount rate. B represents expected net income and D represents a non-monetary use value, while the coefficients $\beta_{1,k}$ and $\beta_{2,k}$ represent preference coefficients placed by agent *k* on B and D; B is given as

$$B = \left[\sum_i p_{i,m} \cdot \left(R_{i,m,t} - C_{i,m,t}\right) + \sum_j \left(I_{j,t} - O_{j,t}\right)\right]$$

where R represents the value derived by agent k from the opportunity i and C is the cost to access it, I represents resources received from a particular network connection j and O represents resources shared to a particular connection j. The parameter $p_{i,m}$ represents the agent's perceived likelihood of gaining access to opportunity i in place m (i.e., getting the job). For opportunities without hard constraints (i.e., fixed number of available slots), this is always 1. For opportunities with hard constraints, agents already in those layers communicate a 1 (i.e., there is a job) to those with whom they interact. This in turn is communicated to other agents via interaction, with agents evaluating their own expectation over time according to:

$$p_{i,m} = p_{a,k} \cdot (1 - f_k)^{t - t_{inform}} + rand() \cdot p_{b,k} \cdot \left(1 - (1 - f_k)^{t - t_{inform}}\right)$$

where t_{inform} is the time step in which the open slot was last reported, f_k is an agent-specific decay rate, and $p_{a,k}$ *and* $p_{b,k}$ are the agent-specific beliefs about their likelihood to get the slot when it is known to be available and not known, respectively. The condition [$\beta_{1,k}$ B+ $\beta_{2,k}$ D < 0] represents a net loss – where costs exceed income plus any other use value or benefit – and the parameter P scales the disutility of losses as per prospect theory.

- Select the best portfolio, across all locations, to assume. If this involves moving to a new location, pay the costs of moving.

SI3.*III.iv.b What are the model parameters, their dimensions and reference values?*

The complete list of agent parameters is given in Table 1. Places in a MIDAS simulation are described by a location in two-dimensional space, and a set of utility layers whose value varies in space and time, but will be the same for each agent accessing them at a given place and time. The environment is further described by a set of costs associated with accessing utility layers (such as licenses that may apply to some or all locations, or machinery, etc.), a set of costs to move between any two locations, and a set of costs to share resources between agents located in any two locations.

Reference values for all of these parameters will be application specific.

SI3.*III.iv.c How were the submodels designed or chosen, and how were they parameterized and then tested?*

The goals of the decision model are described in Section II. Parameterization and testing is application-specific and is not described here.

SI3.*IV* LITERATURE CITED

BBS. 2017. *Yearbook of Agricultural Statistics-2016*. Dhaka, Bangladesh.

Bert, F. E., G. P. Podestá, S. L. Rovere, Á. N. Menéndez, M. North, E. Tatara, C. E. Laciana, E. Weber, and F. R. Toranzo. 2011. An agent based model to simulate structural and land use changes in agricultural systems of the argentine pampas. *Ecological Modelling* 222(19):3486–3499.

Dave, C., C. Eckel, C. Johnson, and C. Rojas. 2010. Eliciting Risk Preferences: When is Simple Better ? *Journal of Risk and Uncertainty* 41(3):219–243.

Egbert, G. D., and S. Y. Erofeeva. 2002. Efficient inverse modeling of barotropic ocean tides. *Journal of Atmospheric and Oceanic Technology* 19(2):183–204.

Feder, G. 1980. Farm Size , Risk Aversion and the Adoption of New Technology under Uncertainty. *Oxford Economics Papers* 32(2):263–283.

Hong, G. 2015. Examining the role of amenities in migration decisions: A structural estimation approach. *Papers in Regional Science* 95(4).

Kahneman, D. 2003. Maps of Bounded Rationality: Psychology for Behavioral Economics. *The American Economic Review* 93(5):1449–1475.

Kopp, R. E., R. M. DeConto, D. A. Bader, C. C. Hay, R. M. Horton, S. Kulp, M. Oppenheimer, D. Pollard, and B. H. Strauss. 2017. Evolving Understanding of Antarctic Ice-Sheet Physics and Ambiguity in Probabilistic Sea-Level Projections. *Earth's Future* 5(12):1217–1233.

Minnesota Population Center. 2015. Integrated Public Use Microdata Series, International: Version 6.4 [dataset]. University of Minnesota, Minneapolis, MN.

Moon, B. 1995. Paradigms in migration research: exploring "moorings" as a schema. *Progress in human geography* 19(4):504–524.

Muis, S., M. Verlaan, H. C. Winsemius, J. C. J. H. Aerts, and P. J. Ward. 2016. A global reanalysis of storm surges and extreme sea levels. *Nature Communications* 7(May).

Müller, B., F. Bohn, G. Dreßler, J. Groeneveld, C. Klassert, R. Martin, M. Schlüter, J. Schulze, H. Weise, and N. Schwarz. 2013. Describing human decisions in agent-based models - ODD+D, an extension of the ODD protocol. *Environmental Modelling and Software* 48:37–48.

Quisumbing, A., and V. Mueller. 2011. How Resilient are Labour Markets to Natural Disasters? The Case of the 1998 Bangladesh Flood. *Journal of Development Studies* 47(12):1954–1971.

Rubenstein, A. 1998. *Modeling Bounded Rationality*. Page (K. G. Persson, editor) *Southern Economic Journal*. MIT University Press, Cambridge, MA.

Schlüter, M., A. Baeza, G. Dressler, K. Frank, J. Groeneveld, W. Jager, M. A. Janssen, R. R. J. Mcallister, B. Müller, K. Orach, N. Schwarz, and N. Wijermans. 2017. A framework for mapping and comparing behavioural theories in models of social-ecological systems. *Ecological Economics* 131:21–35.

Stimson, R. J., and R. McCrea. 2004. A push-pull framework for modelling the relocation of retirees to a retirement village: The Australian experience. *Environment and Planning A* 36(8):1451–1470.

UNDESA. 2013. World Fertility Data 2012.

WHO. 2015. Global Health Observatory data repository.
